# Malabaricane and Isomalabaricane Triterpenoids, Including Their Glycoconjugated Forms

**DOI:** 10.3390/md19060327

**Published:** 2021-06-05

**Authors:** Valentin A. Stonik, Sophia A. Kolesnikova

**Affiliations:** 1 G.B. Elyakov Pacific Institute of Bioorganic Chemistry, Far Eastern Branch of Russian Academy of Sciences, Pr. 100-let Vladivostoku 159, 690022 Vladivostok, Russia; 2 School of Natural Sciences, Far Eastern Federal University, Sukhanova Str. 8, 690000 Vladivostok, Russia

**Keywords:** malabaricanes, isomalabaricanes, triterpenoids, taxonomic distribution, biological activities, biogenesis

## Abstract

In this review, we discuss structural diversity, taxonomic distribution, biological activities, biogenesis, and synthesis of a rare group of terpenoids, the so-called malabaricane and isomalabaricane triterpenoids, as well as some compounds derived from them. Representatives of these groups were found in some higher and lower terrestrial plants, as well as in some fungi, and in a relatively small group of marine sponges. The skeletal systems of malabaricanes and isomalabaricanes are similar to each other, but differ principally in the stereochemistry of their tricyclic core fragments, consisting of two six-membered and one five-membered rings. Evolution of these triterpenoids provides variety of rearranged, oxidized, and glycoconjugated products. These natural compounds have attracted a lot of attention for their biosynthetic origin and biological activity, especially for their extremely high cytotoxicity against tumor cells as well as promising neuroprotective properties in nanomolar concentrations.

## 1. Introduction

**Figure 1:**
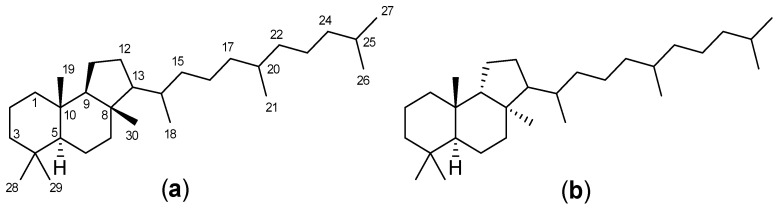
(**a**) Malabaricane skeleton system. (**b**) Isomalabaricane skeleton system.

Triterpenoids are one of the well-known classes of natural products derived by cyclization of either squalene or squalene-2,3-oxide. Common triterpenoids belong to tetra- and/or pentacyclic compounds. Tetracyclic triterpenoids are known as biosynthetic precursors of sterols and other steroids, found in most of the studied eukaryotes. Pentacyclic triterpenoids are more characteristic of prokaryotes and, as glycosides, are widespread in higher plants. [1] The taxonomic distribution of other structural groups of triterpenoids is limited only to distinctive, often small groups of organisms. The malabaricane and isomalabaricane triterpenoids, constructed of tricyclic core systems with long side chains (Figure 1), represent two small groups of terrestrial and marine origin, respectively. [1,2]

## 2. Malabaricanes: Structures, Distribution, Biological Activities, and Biogenesis

### 2.1. Isolation and Structures

**Figure 2:**
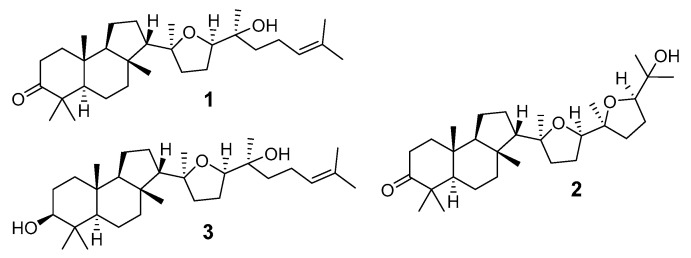
First malabaricane derivatives (**1**–**3**), obtained in 1966–1967.

The first representative of malabaricanes, malabaricol (**l**), was discovered and described as a new triterpenoidal class by Indian chemists from the National Chemical Laboratory in 1967 [3]. It was isolated among series of malabaricanes from the trunk resinous exudate of a tall evergreen rainforest tree *Ailanthus malabarica* (family Simaroubaceae) from India (Figure 2). The general structure of **1** was established by low resolution ^1^H NMR and MS spectra as well as chemical transformations [3], but some stereochemical peculiarities remained undetermined for twelve years until they were studied by X-ray analysis [4]. Related epoxymalabaricol (**2**) and co-isolated malabaricanediol (**3**) were obtained as products of non-enzymatic cyclization of squalene-2,3-oxide [5,6] using stannic chloride and as a result of the oxidation of **2** with Jones reagent [3], respectively. Compound **3** was also synthesized by non-enzymatic cyclization of squalene-2,3-epoxide with picric acid as a catalyst [6]. Later, compounds **1** and **2** were re-isolated from the heartwood of *Ailanthus excelsa* (India) and structurally identified, including the corresponding configurations of their asymmetric centers, by 1D and 2D NMR, IR, and MS techniques [7].

**Figure 3:**
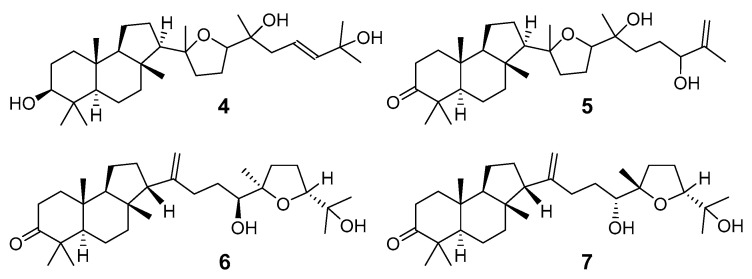
Ailanthusins F (**4**) and G (**5**) from *A. triphysa* and compounds **6**, **7** from *C. echinata*.

It should be noted that *Ailanthus triphysa* (Dennst) (syn. *A. malabarica*) is a deciduous tree common in northern Australia and Thailand, used for a number of therapeutic applications due to anthelmintic, antispasmodic, hypertensive, and other useful properties. It contains metabolites belonging to different chemical classes, including two malabaricane derivatives **4** and **5** (Figure 3). Ailanthusins F (**4**) and G (**5**) were evaluated for cytotoxicity against human cancer cell lines, but none of them displayed cytotoxic effects [8].

Two new malabaricane derivatives **6** and **7** were found in 2002 in the leaves of *Caloncoba echinata,* a plant belonging to the family Flacourtiaceae, distributed in tropical Africa (Figure 3) [9]. Like **1**–**5**, these metabolites contain a 2,2,5-trisubstituted tetrahydrofuran ring as a side chain fragment. The structures of both metabolites were established using 800 MHz NOESY and HMBC correlations.

Detailed studies of the NMR spectra of these and some related compounds were carried out, and it was shown that NOESY data should be used to determine the *cis-* or *trans-* geometry of malabaricanes containing a 2,2,5-trisubstituted tetrahydrofuran ring [10].

**Figure 4:**
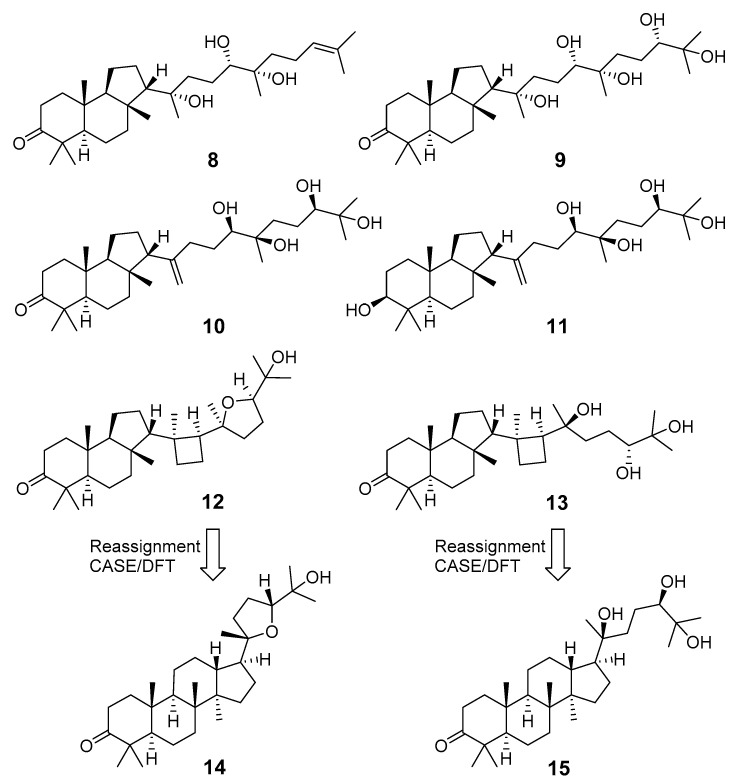
New malabaricanes from *A. malabarica*.

Recent re-investigation of the oleoresin, oozing out of the wounded trunk of *A. malabarica*, which is used in the manufacture of incense sticks in India, reported six new malabaricane triterpenoids **8**–**13** with structures established by extensive 1D and 2D NMR spectroscopy along with triterpenoids **1**–**3** [11]. However, two of these compounds with proposed cyclobutane rings, **12** and **13**, have the same ^13^C NMR data as dammarane triterpenoids ocotillone (**14**) and gardaubryone C (**15**), respectively. Their structural reassignment was confirmed using CASE algorithm and DFT chemical shift predictions and led to the revision of their structures [12]. As a result, only four isolated triterpenoids could be considered as malabaricane derivatives (Figure 4). The CASE expert system is based on well-established set of rules connecting the peculiarities of molecular structures with NMR spectra [13,14]. In that study, it was used together with quantum mechanical calculations, founded on density functional theory (DFT). The HMBC, ^1^H-^1^H COSY, and HSQC-TOCSY spectra were also applied in order to confirm the revised structures **14** and **15**.

**Figure 5:**
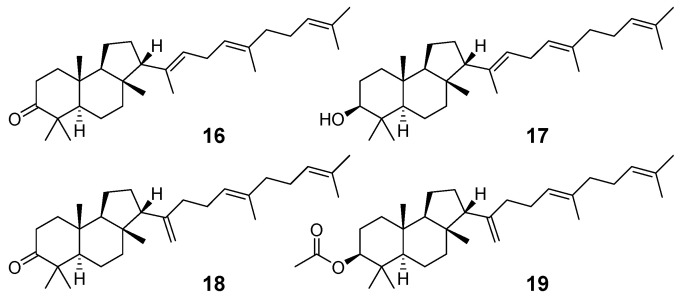
Malabaricane derivatives **16** and **17** from the resin of *B. microphylla* as well as tritepenoids **18** and **19** from *P. santolinoides*.

Malabaricatrienone (**16**) and malabaricatrienol (**17**) (Figure 5) were found in a methanol extract of the resin from the endemic *Bursera microphylla*, the so-called “elephant tree”, widespread in the Sonoran Desert in Mexico. Local people usually steep the plant in alcoholic beverages to treat cold sores and abscessed teeth. These malabaricanes were isolated along with known terpenoids, including *β*-caryophyllene, betulonic acid, and oleanonic acid. The antiproliferative activity of compounds **16** and **17** was evaluated in different murine and human cancer cell lines but they did not show significant effects [15].

**Scheme 1:**
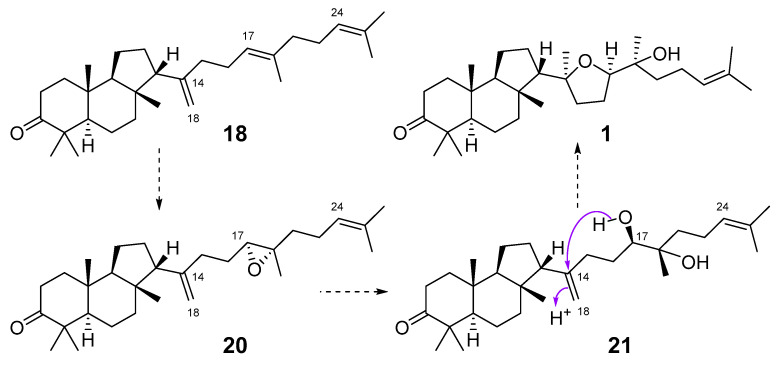
Hypothetical scheme of biotransformation of **18** into malabaricol (**1**).

The roots of *Pyrethrum santolinoides* (syn. *Tanacetum sinaicum*) (family Asteracea), collected from the Sinai Peninsula in Egypt, were studied by German chemists who isolated two malabaricane triterpenoids (**18** and **19**), including a ketone **18**, which is most likely a precursor of malabaricol (**1**) (Figure 5). Malabaricol could be a product of epoxidation of ∆^17^-double bond in **18** led to epoxide **20**, followed by hydrolysis and the formation of tetrahydrofuran ring in **1** as a result of the addition of the secondary hydroxy group to C-14 [16] (Scheme 1).

**Figure 6:**
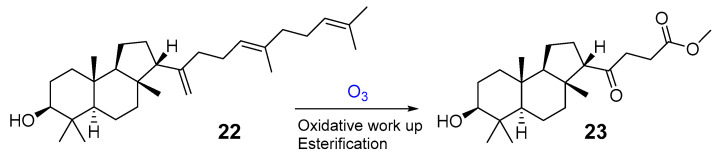
Malabaricane derivative **22** from *P. lentiscus* and its ozonolysis.

The same triterpenoid ketone **18** along with alcohol **22** were isolated from the commercial gum mastic of the pistachio (mastic tree) *Pistacia lentiscus* (Anacaridiaceae) grown in the Mediterranean [17] (Figure 6). The gum mastic is used to improve digestion, oral, and liver health and can be chewed as gum or used in powders, tinctures, and capsules. Moreover, mastic essential oil supports the normal condition of the skin. Structure **22** was confirmed by ozonolysis, which gave dioxoester **23** after oxidative treatment followed by esterification [17] (Figure 6).

**Figure 7:**
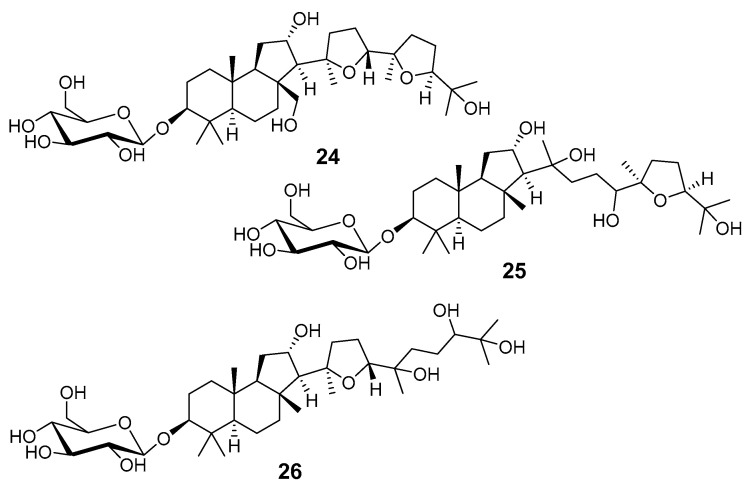
Malabaricane glycosides **24**–**26** from *A. aconcaguensis*.

Unlike other triterpenoid glycosides, glycosides of the malabaricane series were rarely found in the studied plants. Such compounds were isolated from *Adesmia aconcaguensis*, an endemic species from the central Chilean Andes [18]. Three glucopyranosides **24**–**26** (Figure 7) were studied by NMR and FAB MS methods. Glucopyranosyl moieties were indicated by ^1^H and ^13^C NMR spectra as well as by characteristic losses of 162 and 180 mu from the [M + H]^+^ ions in MS. *β*-Orientation of the glucosyl residue in **24** followed from chemical shifts of H-1′ (*δ*_H_ 4.95, *J* = 8.0 Hz) and C-1′ (*δ*_C_ 107.5) in the NMR spectra. Similar chemical shifts were observed in spectra of compounds **25** and **26**. Malabaricane-type aglycones differ from other triterpenoids of this series by the presence of a hydroxy group at C-12. Cytotoxic effects of **24**–**26** against tumor cells were not found.

**Figure 8:**
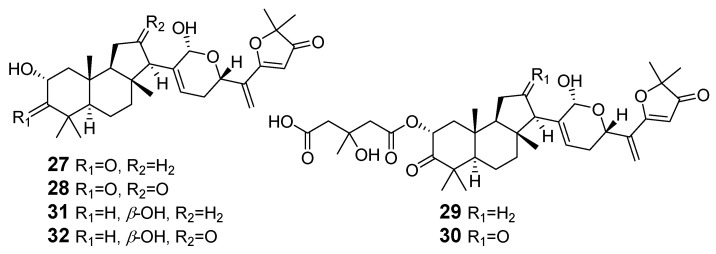
The structures of sodagnitins A–F (**27**–**32**).

Malabaricanes were also found in fungi (order Agaricales) such as *Cortinarius arcuatorum*, *C. sodagnitus*, and others. The cap skin and other parts of these fungi, when exposed to aqueous alkali, gave a color reaction with the formation of an intense inky red tint. Freeze-dried fruit bodies of *C. fulvoincarnatus* and/or *C. arcuotarum* were extracted with ethyl acetate, until the color reaction no longer appeared, and the combined extract was separated by silica gel column chromatography. As a result, six chromogen substances **27**–**32** were isolated and named as sodagnitins A–F [19] (Figure 8). Their structures were established by detailed NMR analysis as well as various mass spectrometry techniques (EI, FAB, and CI).

**Scheme 2:**
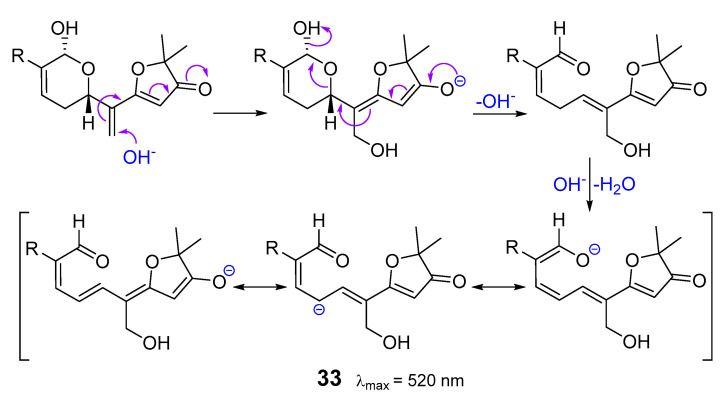
Proposed mechanism for the red color reaction of sodagnitins with alkali.

All the reported compounds belong to malabaricane type and contain chromogenic side chains. The color reaction is triggered by addition of a hydroxide anion to the exomethylene group followed by a fragmentation with concomitant opening of the tetrahydropyran ring. The resulting 1,9-dioxononatriene gives rise the highly delocalized red oxonol-anion **33** with *λ*_max_ = 520 nm (Scheme 2). In support of the proposed scheme, the *λ*_max_ values reported for similar oxonol-anions are in good agreement with those of alkaline solutions of sodagnitins [19].

**Figure 9:**
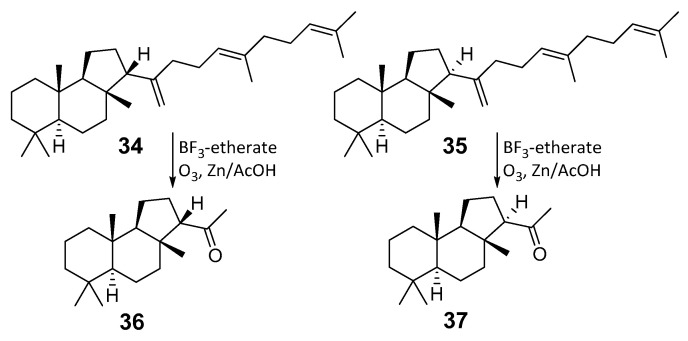
Malabaricanes from the fern *L. microphyllum* var. *obovatum*.

Several triterpenes of malabaricane series were found in some ferns. *Lemmaphyllum microphyllum* var. *obovatum,* collected in Okinawa Prefecture (Japan), belongs to the family Polypodiaceae. It was extracted by hexane, and as a result of column chromatography on silica gel followed by separation using 20% AgNO_3_-Si gel, two new tricyclic hydrocabons 13*β*H-malabaricatriene (**34**) and 13*a*H-malabaricatriene (**35**) were obtained. The report describes the first case of the malabaricane type hydrocarbons isolation in nature (Figure 9) [20]. The stereochemistry of an asymmetric center at C-13 was determined by the treatment of each compound (**34** and **35**) with BF_3_-etherate followed by ozonolysis to obtain the derivatives **36** and **37**, respectively. Among them, the first one was gained previously from malabaricol (**1**) [3,21], while its 13*α*-epimer **37** was known as a product of colysanoxide (**38**) ozonolysis [22]. Comparison of the optical rotation data and CD spectra of the ketones made it possible to establish the absolute configurations of asymmetric centers in **36** and **37** as well as those in malabaricanes **34** and **35**.

**Scheme 3:**
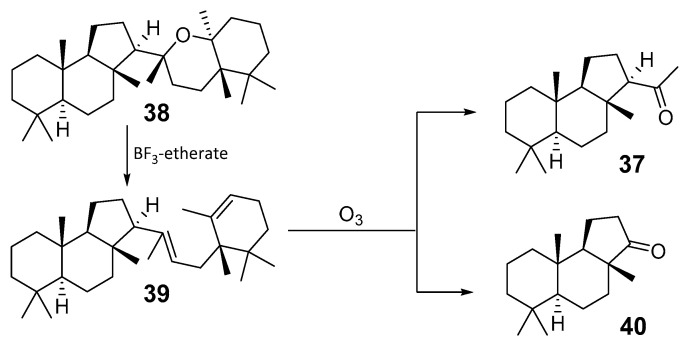
Structure of colysanoxide (**38**) and some of its transformations.

Triterpenoid colysanoxide (**38**), which contains one additional carbocycle in the side chain, when compared with other malabaricanes, was isolated from the Polypodiaceous ferns of the genus *Colysis* from Japan [22]. Its structure **38** was established by spectroscopic methods, accompanied with X-ray analysis, and confirmed by chemical transformations. In fact, **38** was treated with 20% BF_3_-etherate to give unstable diene **39**. The latter was ozonized and converted into methylketone **37** along with a small amount of ketone **40**. The comparison of ORD and CD spectra of **40** with standard compounds allowed the establishment of the absolute configurations in **39** (Scheme 3).

Unexpectedly, the tricyclic hydrocarbon **35**, reported as 17E-13*α*(H)-malabarica-14(27),17,21-triene, was found in the solvent extract of a recent sediment from a sulfur-rich meromictic lake, Cadagno, in Switzerland [23]. It contrasts with other triterpenes, particularly those belonging to the hopane series, which are considered to be among the most abundant natural products on earth and are frequently found in bottom sediments [24]. It is hard to know now from which algal or microbial precursor this rare hydrocarbon may originate. Nevertheless, authors suggested that the biological precursors of the malabaricatriene **35** found in sediments are not ferns. As Lake Cadagno, the Cariaco basin, and the Arabian Sea are sulfur-rich ecosystems, characterized by the presence of anoxic bottom waters and sediments, they proposed that the hydrocarbon may originate from an unknown biological source able to thrive in specific environments [23].

The structural diversity of malabaricanes is limited. Most of them do not contain oxygen atoms additional to 3-hydroxy- or 3-oxo-function; others are oxidized in the side chain. Only a few triterpene glycosides and three compounds, isolated from the fungus *A. aconcaguensis*, were oxidized in the core moiety.

Generally, malabaricane triterpenoids were found in higher plants, ferns, fungi, and bottom sediments (Table 1). It is of special interest that each taxon studied contains only a few species, producing this type of metabolites.

### 2.2. Biogenesis of Malabaricane Triterpenoids and Their Biological Activities

Tetracyclic lanostane and cycloartane derivatives in eukaryotes as well as pentacyclic hopanoids in prokaryotes are the most known classes of polycyclic triterpenoids, which are formed by cyclizations of oxidosqualene and squalene, respectively. Steranes, originated from tetracyclic triterpenoids and hopanes, derived from pentacyclic ones, are frequently found in the sedimentary rocks. These geological records as well as the homology in amino acid sequences of oxidosqualene and squalene cyclases demonstrate that early evolution of triterpenoid cyclases proceeded from common ancestor. Hence, malabaricanoids should be the most ancient polycyclic terpenoids [25].

Mutated squalene–hopene cyclase of *Alicyclobacillus acidocaldarius* with change of Ala for Tyr420 in catalytic cavity cyclizes squalene not only into pentacyclic hopanes and diplopterol, but also into bicyclic polypodatetraenes and tricylic 13α(H)-malabaricatriene [26].

Malabaricane triterpenoids have been tested several times for cytotoxicity against tumor cells, but usually without success; see, for example, [8,15]. However, it was reported very recently that malabaricol (**1**) itself and its numerous synthetic derivatives obtained by condensation with various aldehydes under alkaline conditions show cytotoxic activity against human lung cancer cell lines, comparable with that of standard doxorubicin [27]. Strong antimalarial [9] and/or antibacterial actions [11] were not found except for sodagnitins A (**27**) and C (**29**) from fungi. These malabaricanes proved to be potent inhibitors of bacteria *Bacillus subtilis*, *B. brevis*, and *Nematospora coryli,* and showed strong cytotoxicity against L1210 tumor cells, as well [19].

## 3. Isomalabaricane Triterpenoids. Structures, Properties, Distribution, and Synthesis

### 3.1. Isolation and Structures

**Figure 10:**
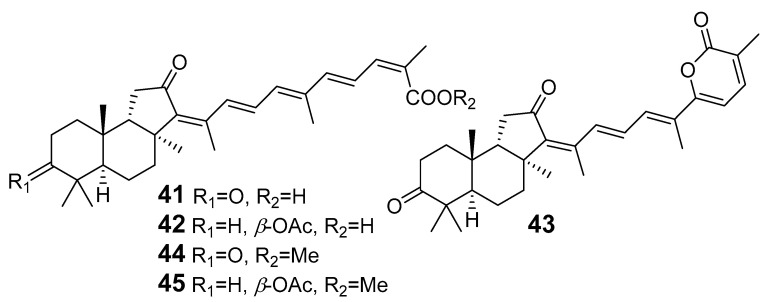
Isomalabaricanes **41**–**43** from the sponge *J. stellifera* and two methyl esters **44**, **45**.

In 1981, Australian scientists from the Roche Research Institute of Marine Pharmacology, together with one researcher from the University of South Pacific (Fiji), described three new triterpenoids **41**–**43** [28], related to malabaricanes, earlier isolated from the tree *A. malabarica* (Figure 10). These triterpenoids were obtained from the extracts of the sponge *Jaspis stellifera* collected on the reef flats of Fiji. The color of this sponge varies from a butter color to an intense bright yellow. Column silica gel chromatography of concentrated dichloromethane extract gave intensely colored yellow fractions, which were immediately treated with diazomethane. Fractions isolated on silica gel column were separated by HPLC to yield two methyl esters **44** and **45** together with compound **43** [28] (Figure 10).

High resolution mass spectrometry established the molecular formula of **44**, while UV spectra with strong bonds at *λ*_max_ = 395 nm and 412 nm showed the presence of a conjugated polyene system. Further NMR analysis and chemical transformations, such as reduction of methyl ester **44** with Zn powder into tetraene **46** and treatment of **44** with ozone followed by dimethylsulfide work up to obtain products **47** and **48**, revealed some structure features of **44**. In addition, tetradeutero-derivative **49** was obtained to simplify analysis of the ^1^H NMR spectrum. In its turn, the hydrogenation of **44** with palladium on charcoal in methanol gave products **50** and **51** along with deoxo-derivative **52**. The latter was studied by CD spectroscopy.

**Scheme 4:**
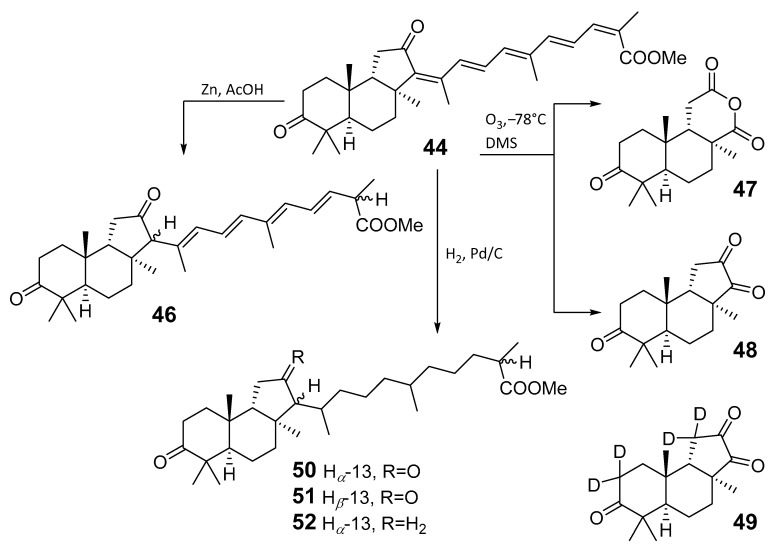
Chemical transformations of methyl ester **44**.

Altogether, the studies of the obtained products by spectroscopic methods established the structures of isolated metabolites, although at that time, the compounds were considered to be malabaricane derivatives with *β*-position of the methyl group at C-8 (Scheme 4).

**Figure 11:**
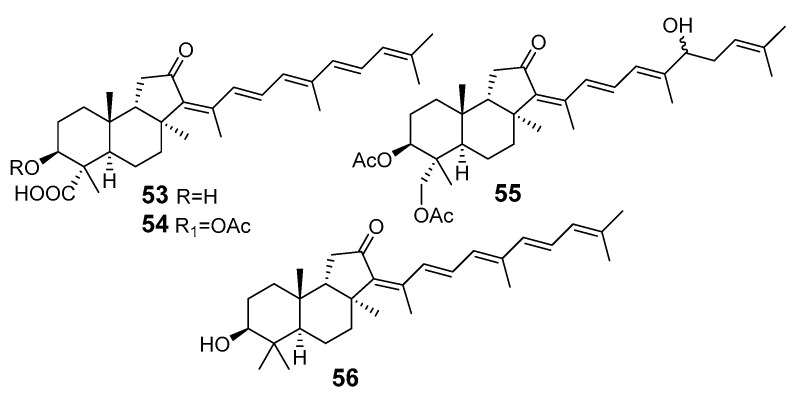
Isomalabaricanes **53**–**56** from a Great Barrier Reef collection of *J. stellifera*.

Similarly, four new triterpenoids **53**–**56** were isolated from a Great Barrier Reef collection of the same sponge and their structures were established by a combination of spectral evidence and chemical degradation [29] (Figure 11). These compounds were also considered to be malabaricane derivatives.

The same compound **43** was soon isolated from the Somalian collection of the sponge *Stelletta* sp. The USA-Italy team used spectroscopic methods in combination with X-ray analysis to elucidate its structure. As a result, it was established that the structure and stereochemistry of **43,** as well as those of other triterpenoids (**41**,**42** and **53**–**56**)**,** isolated from *J. stellifera* collections, should be revised. A new “isomalabaricane” skeleton system with Me*_α_-30* and H*_β_*-9 was proposed for them [30]. Thus, these sponge metabolites differ from malabaricanes in the *trans*–*syn*–*trans* conjunction of the rings in a core system, while malabaricanes possess *trans*–*anti*–*trans* stereochemistry. In more detail, the ring B in isomalabaricane triterpenoids is in the boat conformation in contrast with malabaricanes. These structural conclusions were confirmed by synthesis [31].

It turned out that, despite the limited distribution in nature as substances found in few species of the phylum Porifera (sponges), isomalabaricanes represent a very large and highly biologically active group of secondary metabolites. As a result, these natural compounds attract a great deal of attention from scientists of different countries.

#### 3.1.1. Stellettins

A series of isomalabaricane derivatives, including **43**, was named as stellettins (Table 2). Most of them contain terminal *δ*-lactone ring or carboxyl in a side-chain. Over time, some other related compounds, including *nor*-triterpenoids and rearranged derivatives, were added to this series.

*Stelletta tenuis,* collected off Hainan Island in the South China Sea [32], gave a new unstable isomalabaricane isomer with unsaturated *δ*-lactone, named stellettin A (**57**). This triterpenoid was studied by spectroscopic methods and proved to differ from stellettin B (**43**) in the *E*-geometry of the 13-double bond. Both isomers could be clearly distinguished using ^1^H NMR spectra, as they show a Me-18 signal at *δ*_H_ 2.35 ppm for the *13E*- isomer **57** and at *δ*_H_ 2.07 ppm in the case of the 13*Z-*isomer **43**.

An extract of another representative of the same genus, *Stelletta* sp., collected in Northern Australia, was subjected to bioassay-guided fractionation at the National Cancer Institute (Frederick, MD, USA) to yield four new stellettins C (**58**), D (**59**), E (**60**), and F (**61**), cytotoxic agents against tumor cells with the GI_50_ concentrations in the low-to-mid nanomolar range. The authors noted that stellettins C (**58**) and D (**59**) and all the other pairs of geometric isomers could be separated and purified by HPLC, but each compound rapidly equilibrated to a mixture of geometric isomers upon exposure to light. This behavior of isolated compounds forced the authors to study the cytotoxicity of pairs of interconverting triterpenoids instead of individual compounds. One more isomalabaricane **62** [28], for which the name stellettin G was proposed, was re-isolated from this sponge extract [33]. Structures **58**–**61** were established in the same manner as those of earlier studied metabolites of this series. However, more sensitive spectroscopic methods were used. For example, HR FABMS was applied to deduce molecular formulae.

Stellettins H (**62**) and I (**63**) were found in the extract of the sponge *Rhabdastrella globostellata* from the Philippines together with several related compounds [34], including (−)-stellettin E (**60**), that was surprisingly an optical antipode of a previously reported one [33].

During the study on bioactive triterpenoids from the Fijian marine sponge *R. globostellata* which stabilize the binding of DNA polymerase to DNA, new variants of stellettins, namely J (**64**) and K (**65**), were found [35]. As it was mentioned, some isomalabaricanes can be easily transformed into mixtures of both isomers due to photoisomerization. The report above describes an investigation carried out using defense against sunlight. However, in most cases, such a technique was not used.

Stellettins L (**66**) and M (**67**) from the marine sponge *S. tenuis* from the South China Sea [36] as well as stellettin N (**68**) from the Hainan collection of *Stelletta* sp. sponge [37] increased the series of stellettins having linear polyenic side chain with terminal carboxy group. In the latter case, five known compounds were also found, including 22,23-dihydrostellettin D (**69**) [37], known as metabolite from the sponge *Jaspis* sp. [38]. Analogously, 22,23-dihydrostellettin B (**70**) was reported, among other isomalabaricanes, from Hainanian sponge *Rhabdastrella* aff. *distincta* [39].

Three new unusual compounds, stellettins N (**71**), Q (**72**), and P (**73**), were isolated from the sample of *S. tenuis* and, unfortunately, one of them (**71**) was again named as stellettin N. Isomalabaricane **71** has a hydroxy group at C-13 in the core moiety. The other two compounds **72** and **73** from the same collection contain not only unsaturated *δ*-lactone moiety, but also one hydroxy and one methoxy group in their side chains. These stellettins are evidently distinctive in their structures from most other representatives of this group [40]. In a recent review by Chinese chemists and pharmacologists, a small section on stellettins is given [41].

Structure diversity of stellettins is associated not only with the variety of side chains and, in particular, with the geometry of the 13-double bond, but also with the presence of different functional groups at C-3 in the ring A. There were either *β*-/*α*-hydroxy groups, *β*-acetate, or oxo group found in this position. Instability of some stellettins and related triterpenoids in sunlight brings difficulties to the evaluation of their biological activities and their development as potential drugs. Isolation of individual compounds and their storage could require special protective procedures to prevent photoisomerization of 13-double bond or other specific transformations.

A series of new stellettins **74–79** were described very recently from a marine sponge *Stelletta* sp., collected in the Vietnamese waters. Two of them, **74** and **75**, are rearranged triterpenoids, while the other compounds **76–79** are representatives of *nor*-terpenoidal metabolites. As for any isomalabaricane-related *nor*-terpenoids from sponges, their presence in the sponge extract raises questions and could be explained either by oxidative degradation of isomalabaricane precursors or by enzymatic activity of sponge or even microbial simbionts. In our opinion, the acethylenic derivative, stellettin S (**70**), is the most unusual and unexpected from all these products derived from isomalabaricane-type triterpenoids [42].

**Table 2:** Stellettins.

N	Name	Structure	Collection	Source	Ref.
**57**	Stellettin A	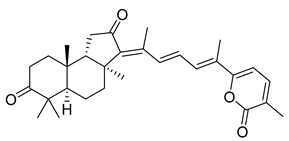	Hainan Island	*S. tenuis,*	[32]
			Cape Wilberforce (Australia)	*Stelletta* sp.	[33]
			Mindanao (Philippines)	*Rhabdastrella globostellata*	[34]
			Hainan Island	*R. aff. distincta*	[39]
			Xisha Island (South China Sea)	*Geodia japonica*	[43]
**43**	Stellettin B	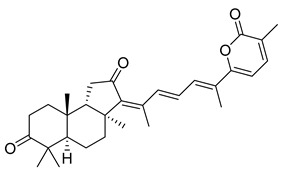	Fiji	*Jaspis stellifera*	[28]
			Great Barrier Reef	*J. stellifera*	[29]
			Somalian waters	*Stelletta* sp.	[30]
			Hainan Island	*S. tenuis*	[32]
			Cape Wilberforce (Australia)	*Stelletta* sp.	[33]
			Mindanao (Philippines)	*R. globostellata*	[34]
			Hainan Island	*R.* aff. *distincta*	[39]
			Xisha Island (South China Sea)	*Geodia japonica*	[43]
**58**	Stellettin C	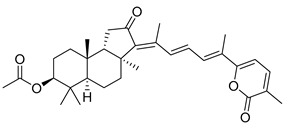	Cape Wilberforce (Australia)	*Stelletta* sp.	[33]
			Mindanao (Philippines)	*R. globostellata*	[34]
			Hainan Island	*R.* aff. *distincta*	[39]
			Hainan Island	*S. tenuis*	[40]
			Hainan Island	*R. globostellata*	[44]
**59**	Stellettin D	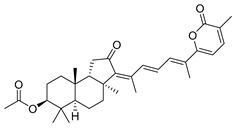	Cape Wilberforce (Australia)	*Stelletta* sp.	[33]
			Mindanao (Philippines)	*R. globostellata*	[34]
			Hainan Island	*Stelletta* sp.	[37]
			Hainan Island	*S. tenuis*	[40]
			Hainan Island	*R. globostellata*	[44]
**60**	Stellettin E	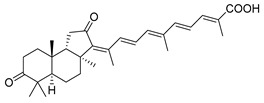	Cape Wilberforce (Australia)	*Stelletta* sp.	[33]
			Mindanao (Philippines)	*R. globostellata*	[34]
			Hainan Island	*R.* aff. *distincta*	[39]
			Hainan Island	*R. globostellata*	[44]
**61**	Stellettin F	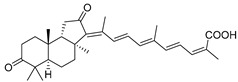	Cape Wilberforce (Australia)	*Stelletta* sp.	[33]
**41**	Stellettin G	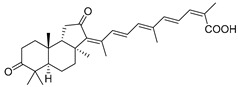	Fiji	*J. stellifera*	[28]
			Cape Wilberforce (Australia)	*Stelletta* sp.	[33]
			Hainan Island	*Stelletta* sp.	[37]
**62**	Stellettin H	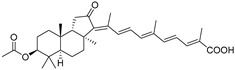	Mindanao (Philippines)	*R. globostellata*	[34]
			Hainan Island	*Stelletta* sp.	[37]
**63**	Stellettin I	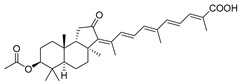	Mindanao (Philippines)	*R. globostellata*	[34]
**64**	Stellettin J	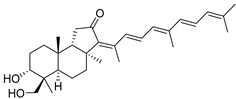	Fiji	*R. globostellata*	[35]
**65**	Stellettin K	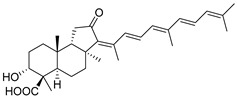	Fiji	*R. globostellata*	[35]
**66**	Stellettin L	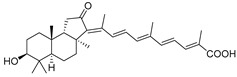	Hainan Island	*S. tenuis*	[36]
			Hainan Island	*R.* aff. *distincta*	[45]
**67**	Stellettin M	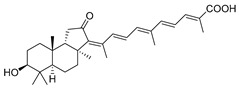	Hainan Island	*S. tenuis*	[36]
			Hainan Island	*R.* aff. *distincta*	[45]
**68**	Stellettin N	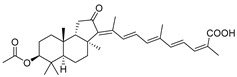	Hainan Island	*Stelletta s*p.	[37]
**69**	22,23-Dihydrostellettin D	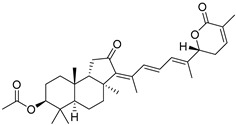	Hainan Island	*Stelletta s*p.	[37]
			South China Sea,	*Jaspis* sp.	[38]
			South China Sea	*J. stellifera*	[46]
**70**	22,23-Dihydrostellettin B	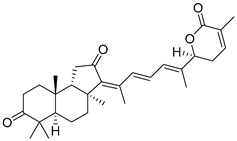	Hainan Island	*Rhabdastrella* aff. *distincta*	[39]
**71**	Stellettin N	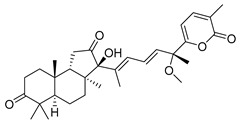	Hainan Island	*S. tenuis*	[40]
**72**	Stellettin O	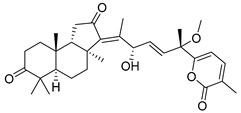	Hainan Island	*S. tenuis*	[40]
**73**	Stellettin P	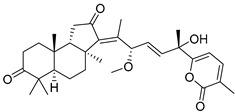	Hainan Island	*S. tenuis*	[40]
**74**	Stellettin Q	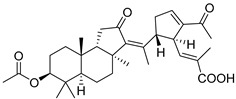	Cham Island (Vietnam)	*Stelletta* sp.	[42]
**75**	Stellettin R	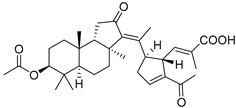	Cham Island (Vietnam)	*Stelletta* sp.	[42]
**76**	Stellettin S	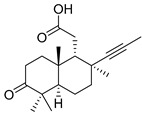	Cham Island (Vietnam)	*Stelletta* sp.	[42]
**77**	Stellettin T	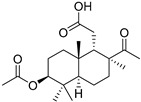	Cham Island (Vietnam)	*Stelletta* sp.	[42]
**78**	Stellettin U	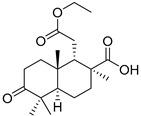	Cham Island (Vietnam)	*Stelletta* sp.	[42]
**79**	Stellettin V	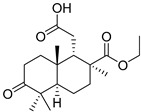	Cham Island (Vietnam)	*Stelletta* sp.	[42]

#### 3.1.2. Stelliferins

Stelliferins are known as another structural group of isomalabaricane triterpenoids, which usually have oxygenated side chains with various functional groups, such as hydroxy or oxo functions (Table 3).

Stelliferins A–F (**80**–**85**) from the Okinawan collection of the sponge *Jaspis stellifera* were isolated by column chromatography on silica gel and sephadex LH-20 followed by reversed-phase HPLC [47]. Structures of these compounds were established by spectroscopic methods. Stereochemistry at C-22 and other stereochemical peculiarities were determined using Mosher’s procedure and NOESY experiments.

Three isomalabaricane triterpenoids **86**–**88** from the sponge *Jaspis* sp. collected near the Tonga archipelago included stelliferin G (**86**) and hydroxylated stelliferins A (**87**) and E (**88**), containing hydroxy group attached to C-29. Hence, they have additional functional groups both in the core moiety and in the side chains, in contrast to most stellettins.

It is also of special interest that many triterpenoids of this series contain acetate groups in the ring A. To confirm that the acetate groups in stelliferins **86**–**88** were not artificial, due to the use of ethyl acetate, Meragelman et al. excluded this solvent from the isolation procedures. The same compounds **87** and **88**, obtained without ethyl acetate, supported their natural origin. In their experimental work, these authors also paid attention to the instability of some isomalabaricane triterpenoids and provided their protection by wrapping all glassware in aluminum foil. Moreover, before bioassays, they evaluated the dynamic of sunlight-induced interconversion of corresponding pairs of *Z*/*E*-isomers under exposure to ambient light. The isomeric mixtures showed antiproliferative activity against melanoma cells (MALME-3M) [48].

For the first time, 3-*epi*-29-hydroxystelliferin A (**89**), along with two other closely related triterpenoids **90** and **91,** were isolated from *Stelletta globostellata* by Japanese chemists, who collected this sponge near Mage-jima Island, Japan [49]. These isomalabaricanes contain 3*α* or 3*β-*hydroxy group together with 29-hydroxyl in a ring A of the core part of their structures.

Another scientific group from Japan found a series of structurally unique stelliferins J-N (**92**–**96**) with the side chains containing two hydroxy groups and, in the case of **92** and **93**, additional oxo function. Three of these isomalabaricanes have five-membered cycles in their side-chains. To establish complex stereostructures with three additional asymmetric centers, the authors had to apply a modified Mosher’s method and exciton chirality method [50].

In addition, stelliferins **97**–**100** were mentioned as components of the active *Z*/*E*-isomeric mixtures of corresponding isomalabaricanes from *Jaspis* sp. collected near the Tonga archipelago [48,51].

The first isomalabaricane glycoside, stelliferin riboside (**101**) with ribopyranose attached to C-22 in its side chain, as well as known stellettins A (**57**) and B (**43**), were isolated from the Fijian sponge *Geodia globostellifera* by scientists from Scotland. The sponge was collected off Vanua Levu Island (Fiji) [52].

Later, two new moderately cytotoxic triterpene glycosides of stelliferin series, **102** and **103**, along with twelve closely related triterpenoids, were found in the sponge *Rhabdastrella globostellata* collected off Indonesia [53]. The configuration of C-22 remains undetermined for all stelliferin ribosides. However, in both reports above, it was noted that in contrast with other isomalabaricanes, having conjugated linear side-chains, congeners with a keto group at C-15 or a ribose substituent at C-22, as in derivatives **100**–**102**, seem to be more stable in maintaining their C-13,C-14-geometry [52,53].

It is interesting that contrary to plant malabaricane glycosides **24**–**26** from *A. aconcaguensis* [18] with 3-*β*-glucosyl residues, three glycosides of stelliferin series, found in Indonesian *R. globostellata*, contain ribose substituents at C-22 in the side-chains. Glycocongugates are rare in both malabaricanes and isomalabaricanes.

**Table 3:** Stelliferins.

N	Name	Structure	Collection	Source	Ref.
**80**	Stelliferin A	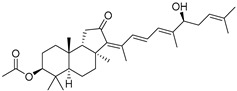	Isigaki Island (Japan)	*Jaspis stellifera*	[47]
			Mage-jima Island (Japan)	*Stelletta globostellata*	[49]
**81**	Stelliferin B	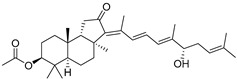	Isigaki Island (Japan)	*J. stellifera*	[47]
**82**	Stelliferin C	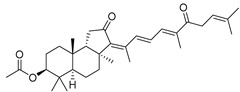	Isigaki Island (Japan)	*J. stellifera*	[47]
**83**	Stelliferin D	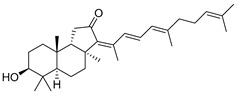	Isigaki Island (Japan)	*J. stellifera*	[47]
			Mage-jima Island (Japan)	*S. globostellata*	[49]
**84**	Stelliferin E	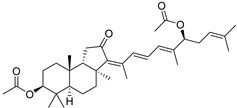	Isigaki Island (Japan)	*J. stellifera*	[47]
**85**	Stelliferin F	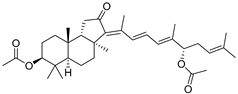	Isigaki Island (Japan)	*J. stellifera*	[47]
**86**	Stelliferin G	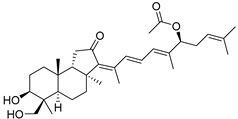	Tonga (South Pacific)	*Jaspis* sp.	[48]
**87**	29-Hydroxy- stelliferin A	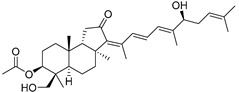	Tonga (South Pacific)	*Jaspis* sp.	[48]
**88**	29-Hydroxy- stelliferin E	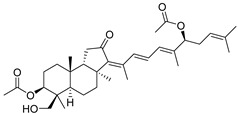	Tonga (South Pacific)	*Jaspis* sp.	[48]
**89**	3-*Epi*-29- Hydroxy- stelliferin A	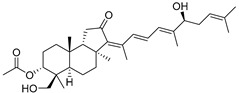	Mage-jima Island (Japan)	*S. globostellata*	[49]
**90**	3-*Epi*-29- Hydroxy- stelliferin E	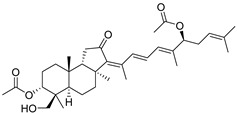	Mage-jima Island (Japan)	*S. globostellata*	[49]
			Tonga (South Pacific)	*Jaspis* sp.	[48]
**91**	29-Hydroxy- stelliferin D	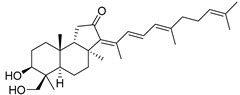	Mage-jima Island (Japan)	*S. globostellata*	[49]
**92**	Stelliferin J	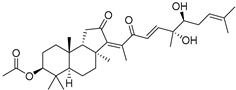	Ishigaki (Japan)	*Rhabdastrella* cf. *globostellata*	[50]
**93**	Stelliferin K	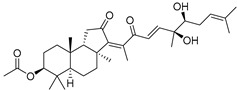	Ishigaki (Japan)	*R.* cf. *globostellata*	[50]
**94**	Stelliferin L	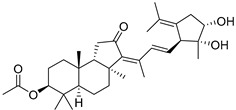	Ishigaki (Japan)	*R.* cf. *globostellata*	[50]
**95**	Stelliferin M	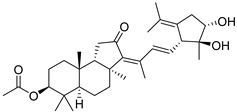	Ishigaki (Japan)	*R.* cf. *globostellata*	[50]
**96**	Stelliferin N	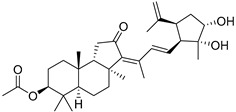	Ishigaki (Japan)	*R.* cf. *globostellata*	[50]
**97**	29-Hydroxy- stelliferin B	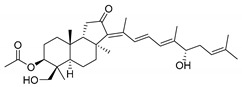	Tonga (South Pacific)	*Jaspis* sp.	[48,51]
**98**	13(*E*)-29- Hydroxy- stelliferin E	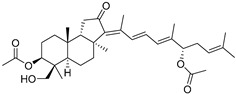	Tonga (South Pacific)	*Jaspis* sp.	[48]
**99**	3-*epi*-29- Hydroxy- stelliferin E	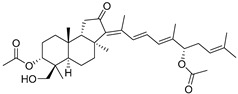	Tonga (South Pacific)	*Jaspis* sp.	[48]
**100**	13(*E*)- stelliferin G	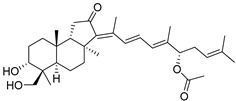	Tonga (South Pacific)	*Jaspis* sp.	[48]
**101**	Stelliferin riboside	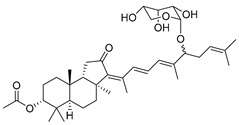	Vanua Levu Island (Fiji)	*Geodia* *Globostellifera*	[52]
			Kapoposang Island (Indonesia)	*R. globostellata*	[53]
**102**	3-O-Deacetyl-13(*Z*)- stelliferin riboside	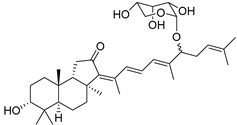	Kapoposang Island (Indonesia)	*R. globostellata*	[53]
**103**	13(*E*)- Stelliferin riboside	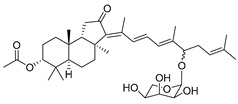	Kapoposang Island (Indonesia)	*R. globostellata*	[53]

#### 3.1.3. Globostellatic Acids

During long-term studies on marine sponges and their bioactive metabolites, Prof. Nobuhiro Fusetani and his team discovered many diverse bioactive compounds. In 1996, they found that methanolic extract of the marine sponge *Stelletta globostellata* collected off Mage Island near Kagoshima (Japan) showed potent cytotoxic activity against tumor cells. Globostellatic acids isolated by them represent the third numerous series of isomalabaricane sponge triterpenoids (Table 4). The first representatives of this group, globostellatic acids A–D (**104**–**107**) [54], were isolated in the form of sodium salts of the corresponding carboxylic acids. The presence of a carboxyl group at C-4 in the tricyclic core is a characteristic structural peculiarity of these compounds. Another characteristic feature is the 3*α*-oriented hydroxy or acetoxy groups at C-3.

Globostellatic acid E (**108**) as a methyl ester was reported by Zampella et al. from the marine sponge *Jaspis* sp. [55].

Many other globostellatic acids **109**–**116** were isolated from the sponge *Rhabdastrella globostellata*, collected from Kapoposang Island (Indonesia) [53]. Their structures and biological sources are given also in the review article [56]. Since Fouad et al. had at their disposal the pair of *Z*/*E*-isomers **107** and **110**, they were able to compare their NMR signals and propose 13*E*-configuration for globostellatic acid D (**107**), instead of previously reported 13*Z*-geometry [53].

Six globostellatic acids methyl esters were obtained from the extract of the marine sponge *R. globostellata,* collected from Sulawesi Island, Indonesia [57]. In this bioassay-guided research, the active fraction of the sponge was converted into methyl esters with TMS–diazomethane treatment and separated by reversed-phase HPLC. All triterpenoids **117**–**122** discovered from this collection have a 3*α*-acetoxy group and a 4*β*-carboxyl function, like many other globostellatic acids.

**Table 4:** Globostellatic acids.

N	Name	Structure	Collection	Source	Ref.
**104**	Globostellatic acid A	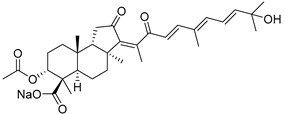	Mage Island (Japan)	*Stelletta* *globostellata*	[54]
			Kapoposang Island (Indonesia)	*Rhabdastrella globostellata*	[53]
**105**	Globostellatic acid B	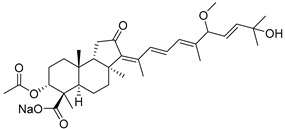	Mage Island (Japan)	*S. globostellata*	[54]
			Vanuatu Islands	*Jaspis* sp.	[55]
**106**	Globostellatic acid C	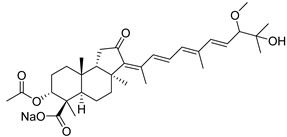	Mage Island (Japan)	*S. globostellata*	[54]
			Vanuatu Islands	*Jaspis* sp.	[55]
**107**	Globostellatic acid D	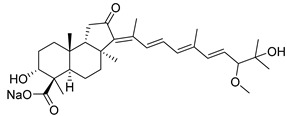	Mage Island (Japan)	*S. globostellata*	[54]
			Kapoposang Island (Indonesia)	*R. globostellata*	[53]
**108**	Globostellatic acid E methyl ester	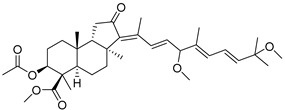	Vanuatu Islands	*Jaspis* sp.	[55]
**109**	Globostellatic acid F	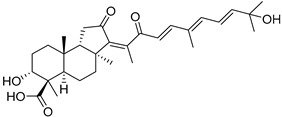	Kapoposang Island (Indonesia)	*R. globostellata*	[53]
**110**	Globostellatic acid G	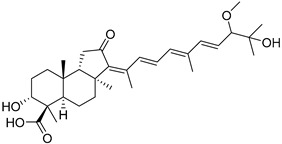	Kapoposang Island (Indonesia)	*R. globostellata*	[53]
**111**	Globostellatic acid H	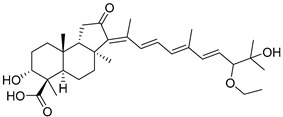	Kapoposang Island (Indonesia)	*R. globostellata*	[53]
**112**	Globostellatic acid I	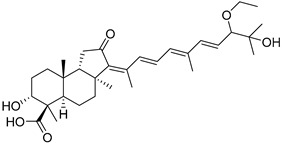	Kapoposang Island (Indonesia)	*R. globostellata*	[53]
**113**	Globostellatic acid J	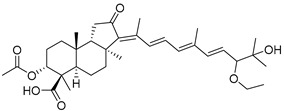	Kapoposang Island (Indonesia)	*R. globostellata*	[53]
**114**	Globostellatic acid K	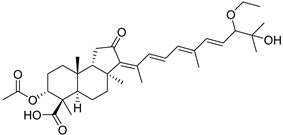	Kapoposang Island (Indonesia)	*R. globostellata*	[53]
**115**	Globostellatic acid L	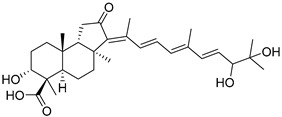	Kapoposang Island (Indonesia)	*R. globostellata*	[53]
**116**	Globostellatic acid M	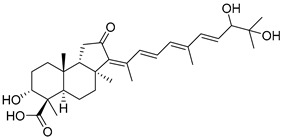	Kapoposang Island (Indonesia)	*R. globostellata*	[53]
**117**	13*Z*,17*Z*- Globostellatic acid X methyl ester	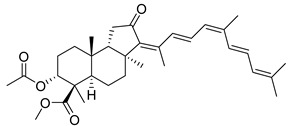	Sulawesi Island (Indonesia)	*R. globostellata*	[57]
**118**	13*Z*,17*E*- Globostellatic acid X methyl ester	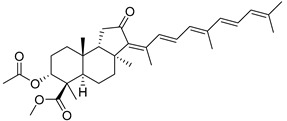	Sulawesi Island (Indonesia)	*R. globostellata*	[57]
**119**	13*E*,17*Z*- Globostellatic acid X methyl ester	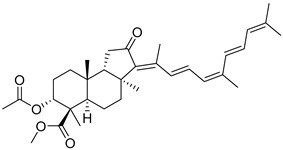	Sulawesi Island (Indonesia)	*R. globostellata*	[57]
**120**	13*E*,17*E*- Globostellatic acid X methyl ester	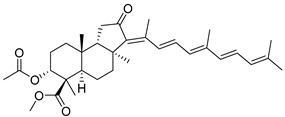	Sulawesi Island (Indonesia)	*R. globostellata*	[57]
**121**	Globostellatic acid F methyl ester	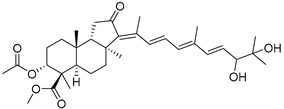	Sulawesi Island (Indonesia)	*R. globostellata*	[57]
**122**	13*E*-Globostellatic acid B methyl ester	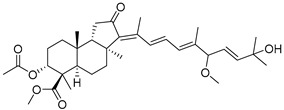	Sulawesi Island (Indonesia)	*R. globostellata*	[57]

#### 3.1.4. Other Isomalabaricane Triterpenoids and *Nor*-Triterpenoids

**Figure 12:**
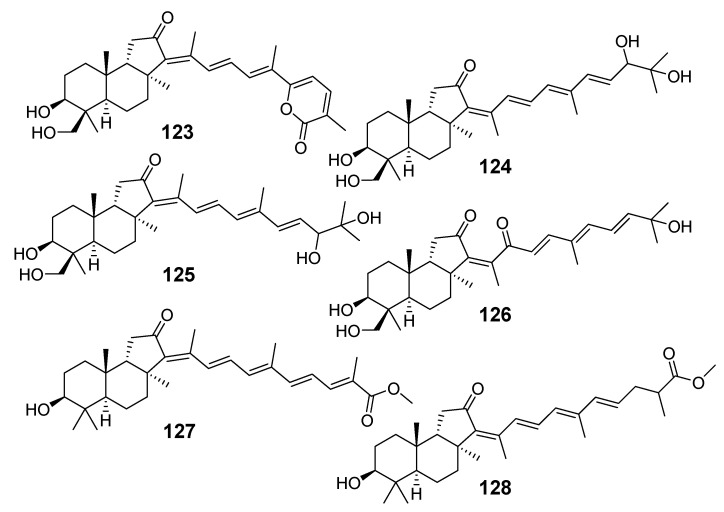
Rhabdastrellins A–F (**123**–**128**) from *R.* aff. *distincta*.

Rhabdastrellins A–F (**123**–**128**) from the sponge *Rhabdastrella* aff. *distincta* from the coral reef of Hainan are isomalabaricane triterpenoids structurally resembling some stellettins. Moreover, it was shown that compounds **127** and **128** are methyl esters of stellettins L (**66**) and M (**67**) [45] (Figure 12).

**Figure 13:**
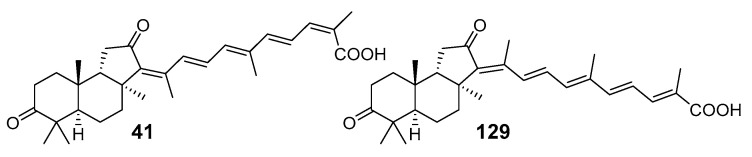
Structures of stellettin G (**41**) and rhabdastrellic acid A (**129**).

Compound **41** was isolated in 1981 [28] as one of the first representatives of isomalabaricanes. Its structural elucidation enquired multiple chemical transformations. However, the stereochemistry of **41** was subsequently revised by McCabe’s group based on the results of a single crystal X-ray study carried out for the related compound **43** [30]. Sixteen years later, one of its isomers was described by the scientists from the Guangzhou Institute of Chemistry and Shanghai Institute of Organic Chemistry. They isolated it from the sponge *R. globostellata* collected in the South China Sea and named as rhabdastrellic acid A (**129**) [58]. Two compounds, stellettin G (**41**) and rhabdastrellic acid A (**129**), differ from each other in stereochemistry of 13- and 24-double bonds (Figure 13).

**Figure 14:**
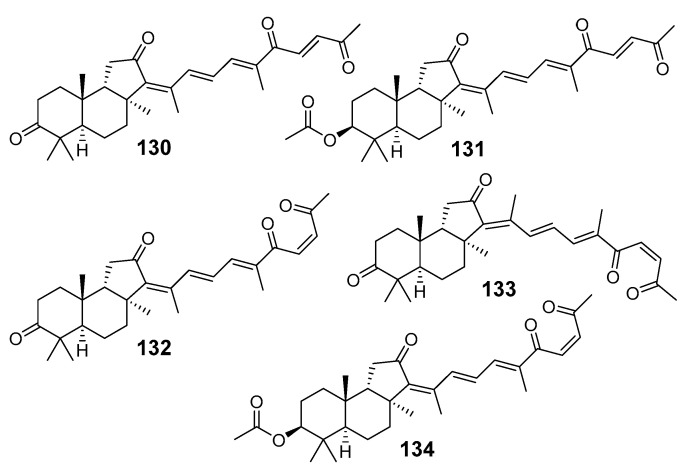
Geoditins **130** and **131** from *G. japonica* and isogeoditins **132**–**134** from *R.* aff. *distincta*.

Numerous *nor*-triterpenoids, derived from isomalabaricanes and containing fewer than 30 carbon atoms, were found in some sponges. For example, geoditins A (**130**) and B (**131**) (Figure 14) were isolated without protection against light from the *Geodia japonica* collected off Xisha Island (South China Sea) and structurally elucidated by spectroscopic methods as new isomalabaricane-type *nor*-triterpenoids [43]. It could be presumed that these compounds originated from isomalabaricanes similar to rhabdastrellic acid or stellettins via loss of the terminal methyl group in the side-chain, likely as a result of decarboxylation, followed by oxidation. Isomeric isogeoditins (**132**–**134**) were isolated from the sponge *R.* aff. *distincta* collected off an inner coral reef at Hainan Island [39].

**Figure 15:**
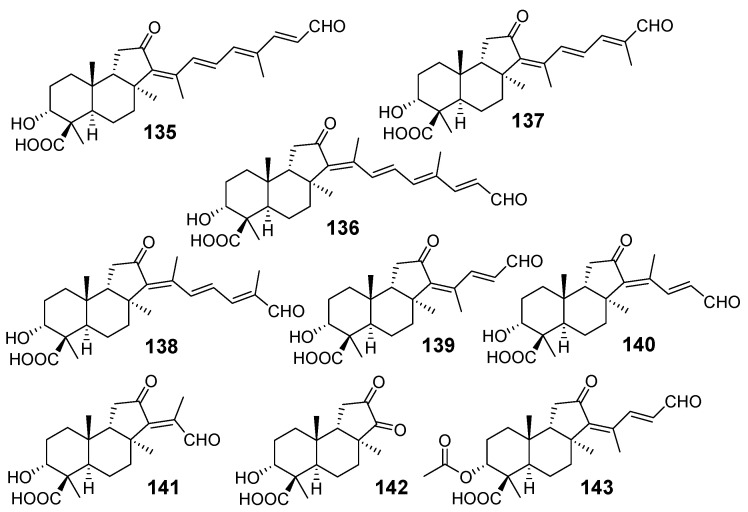
Jaspiferals A–G (**135**–**141**) from *J. stellifera* and acetyljaspiferal E (**143**) from *R. globostellata*.

Structurally unique *nor*-triterpenoids were isolated from the Okinawan marine sponge *J. stellifera* collected off Ishigaki Island (Japan). Jaspiferals A–G (**135**–**141**) lost four to ten carbon atoms in their skeleton systems in comparison with classical triterpenoids. In addition, they are oxidized and bear carboxy and aldehyde groups. Finally, jaspiferals have *α*-oriented hydroxy group at C-3, in contrast with many other isomalabaricanes (Figure 15) [59].

Pairs of compounds **135** and **136**, **137** and **138,** and **139** and **140** were isolated as 1:1 mixtures. Jaspiferal G (**141**) was obtained as a pure compound. The above-mentioned mixtures were converted into methyl esters with diazomethane and then separated by HPLC using a phenyl-group bonded silica gel column. The *trans*–*syn*–*trans* geometry of A–C rings in jaspiferals was proposed by comparison of their ^13^C chemical shifts with those of known stelliferins and also by NOESY experiments. In detail, stereochemistry of the core moiety was determined by the NOE experiments carried out on native jaspiferals A (**135**) and B (**136**) as well as on the ozonolysis product **142**, obtained from methyl esters of some of isolated metabolites. Mosher’s NMR procedure was used for consideration of the absolute configuration at C-3 [59]. Acetyljaspiferal E (**143**) was found from the marine sponge *R. globostellata* from sea waters of Indonesia [57].

**Figure 16:**
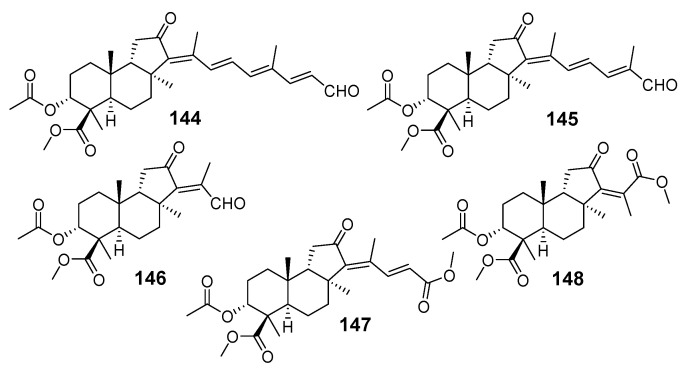
Isomalabaricane *nor*-triterpenoids **144**–**148** from *Jaspis* sp. (Vanuatu Islands).

*Nor*-triterpenoids **144**–**148**, along with the above globostellatic acids B (**105**), C (**106**), and methyl ether of globostellatic acid E (**108**), were obtained from *Jaspis* sp. (Vanuatu Islands) using flesh-chromatography of the sponge extract on a silica gel column followed by methylation with diazomethane and normal phase HPLC. Structures of methyl esters of 3-*O*-acetyljaspiferals B (**144**), D (**145**), G (**146**), and dimethyl esters of jaspiferoic acids B (**147**) and A (**148**) were also established by spectroscopic methods and by comparison with previously known compounds (Figure 16) [55]. A clear structural similarity can be traced between the last two groups of compounds, since compounds **147** and **148** may be considered as the products of oxidation of corresponding jaspiferals.

**Scheme 5:**
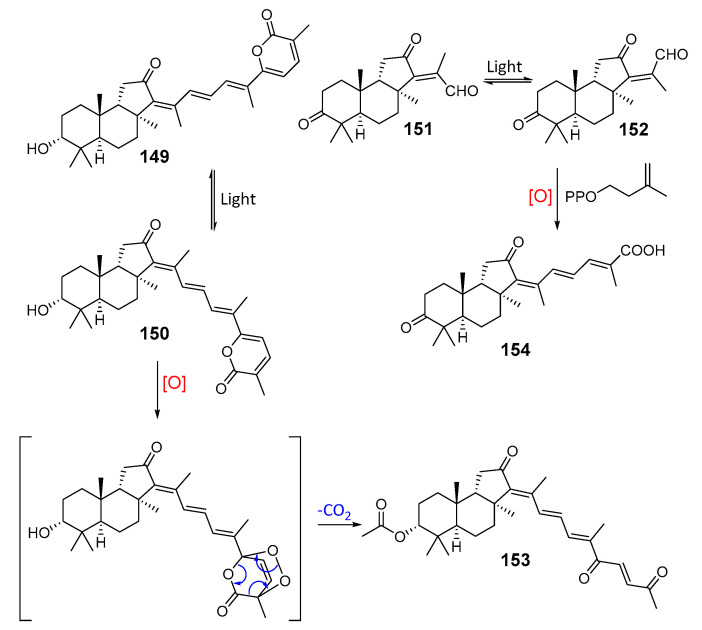
Structures of jaspolides A–F (**149**–**154**) and hypothetical scheme of their interconversion.

Another series of related triterpenoids, jaspolides A–F (**149**–**154**) from the sponge *Jaspis* sp. collected on reefs in Sanya (Hainan Island), included both classical triterpenoids and *nor*-triterpenoids. Structures of jaspolides, isolated from methanol extract of the frozen sponge, were studied using extensive spectroscopic data. Under the light, compounds of this type interconverted from the 13*Z-*forms into the 13*E*-isomers, like many other isomalabaricanes. It was proposed that some of them could be formed due to addition of active isoprene units to *nor*-triterpenoids [60]. Probably, shortened side-chain *nor*-triterpenoids with 13*E*-configuration, for example jaspolide D (**152**), could give metabolites with a longer side-chain, such as jaspolide F (**154**), when reacted with isopropenyl pyrophosphate. On the other hand, in the case of compounds **149** and **150** with longer side-chains, the degradation in the light followed by oxidation and decarboxylation can lead to the shortening of their side-chains with formation of the *nor*-triterpenoid jaspolide E (**153**). However, suggested transformations remain hypothetical and require further confirmation (Scheme 5).

**Scheme 6:**
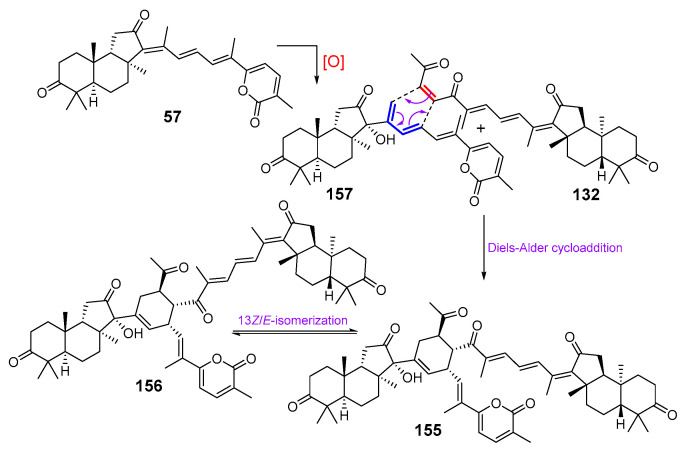
Hypothetical biogenetic pathway for jaspolides G (**155**) and H (**156**).

Two unique isomalabaricane-based dimers, jaspolides G (**155**) and H (**156**), were isolated from the same Hainanian collection of the sponge *Jaspis* sp. [61]. The authors explained a possible origin of these compounds by Diels–Alder dimerization of two other isomalabaricane triterpenoids. One of them is a hypothetical product **157**, likely derived as a result of oxidation of known stellettin A (**57**), and another one is isogeoditin A (**132**) (Scheme 6). The proposed pathway suggested that oxidized stellettin A (**157**) might be a precursor of upper part while isogeoditin A (**132**) formed another moiety of these dimers. Thus, the Diels–Alder cycloaddition with the participation of the 23-double bond of isogeoditin A and the 15- and 14(18)-double bonds of the intermediate **157** results in the formation of a cyclohexene fragment. The structures of these unusual dimers **155** and **156** were elucidated on the basis of spectroscopic data, particularly HMBC and NOESY spectra. Jaspolides G (**155**) and H (**156**) are geometrical isomers differing from each other in stereochemistry of 13-double bond due to the well-known 13*Z*/*E*-isomerization of isomalabaricane triterpenoids.

**Figure 17:**
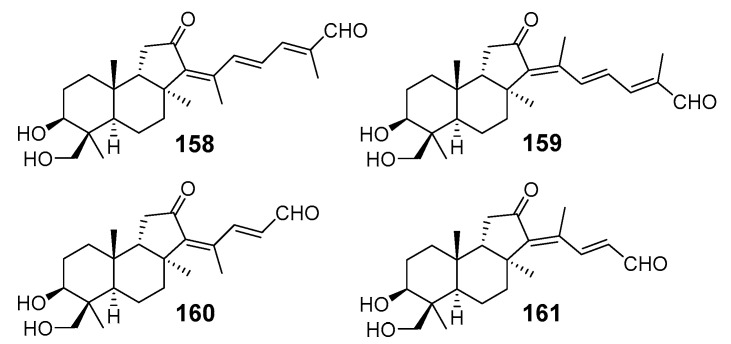
Structures of aurorals A–D (**158**–**161**) from *R. globostellata*.

Cytotoxic *nor*-triterpenoids aurorals A–D (**158**–**161**) (Figure 17) were isolated from the sponge *R. globostellata* by Bourguet-Kondracki et al. from France. At the first stage of the isolation procedure, the sponge was lyophilized and extracted with dichloromethane without protection against sunlight. Formally, some aurorals could be considered as sesterterpenoids because they consist of five isoprene units. All aurorals A–D have a *β*-hydroxymethyl group attached to C-4 in the isomalabaricane core as a characteristic structure feature [62]. Based on the findings of the closely related isomalabaricane terpenoids from the marine sponges *Jaspis stellifera* [28,29,47,59], *Stelletta* sp. [30], *S. tenuis* [32], S. *globostellata* [54], and *R. globostellata* [58], the authors proposed to reassign *J. stellifera* as *Rhabdastrella* sp. [62].

**Scheme 7:**
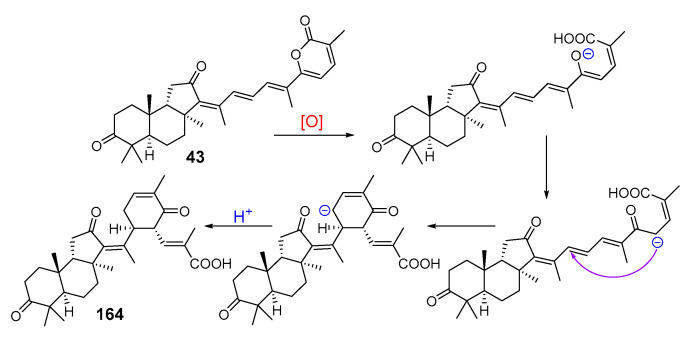
Proposed biogenesis of jaspiferin C (**164**).

**Figure 18:**
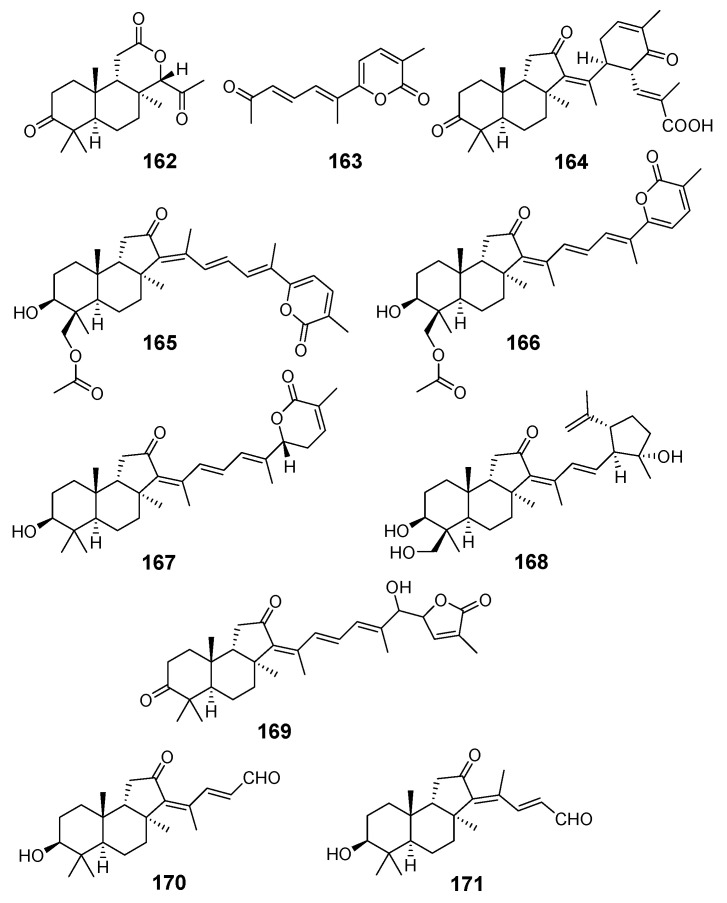
Structures of jaspiferins A–J (**162**–**171**) from two samples of *J. stellifera*.

Two sponge samples from the South China Sea, identified as *Jaspis stellifera,* contained another series of isomalabaricane-type *nor*-triterpenoids, the so-called jaspiferins. However, jaspiferins A (**162**) and B (**163**) from the sponge, collected at Guangdong (South China Sea), actually have no tricarbocyclic isomalabaricane core [63]. Nevertheless, jaspiferins C-F (**164**–**167**) [64], jaspiferin G (**168**) [46], as well as recent jaspiferins H-J (**169**–**171**) [65] were isolated from another sample of *J. stellifera* as new members of the isomalabaricane triterpenoid family (Figure 18). With that, jaspiferin C (**164**), containing side-chain six-membered cycle as an unusual structure feature, was presumably formed as a result of lactone ring opening in stellettin B (**43**) followed by conversions shown in Scheme 7 [64]. The structures of all isolated compounds were established by extensive application of spectroscopic methods.

**Figure 19:**
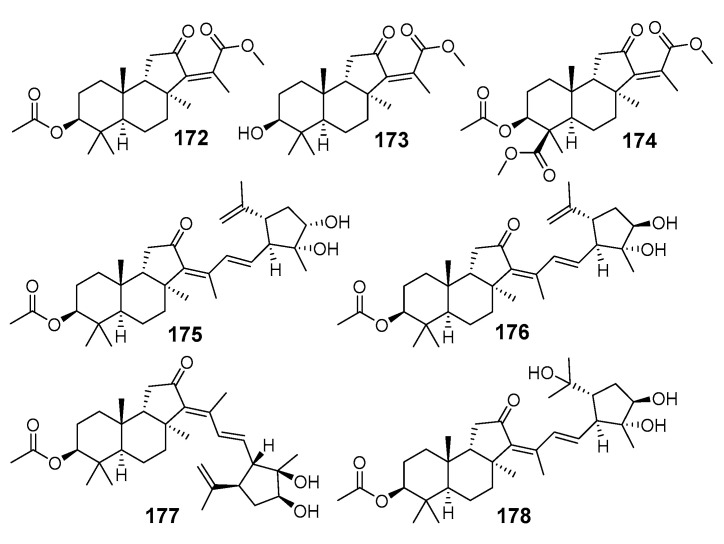
Structures of rhabdastins A–G (**172**–**178**) from *R. globostellata*.

Rhabdastins A–G (**172**–**178**) were isolated from the dichloromethane soluble materials of the *R. globostellata* methanol extract, cytotoxic against tumor cells (Figure 19) [66]. The studied sample was collected in the area of Amami Oshima Island, Japan. All seven rhabdastins obtained were less toxic, some of them even inactive, when compared with initial dichloromethane fraction. Four of these compounds, **175**–**178**, contain in their side-chains cyclopentane moiety similar to that of the aforementioned jaspiferin G (**168**) [46]. Structures of **172**–**178** were determined using NMR and MS spectroscopic methods along with application of X-ray analysis.

**Scheme 8:**
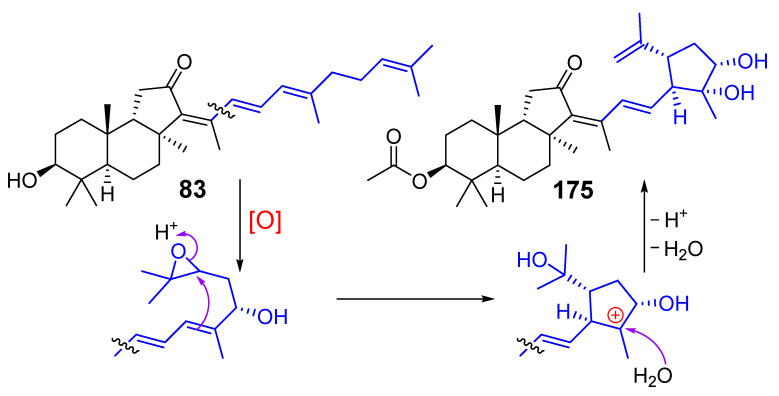
Probable formation of rhabdastin D (**175**) from stelliferin D (**83**).

Rhabdastins D (**175**) and E (**170**) were shown to induce apoptosis in HL-60 tumor cells in the dose of 10 µM. It was also suggested that five-membered rings in rhabdastins might be formed from various stelliferins by oxidation of the terminal double bond to form intermediate epoxides followed by a π-attack of the 17(20)-double bond and epoxide ring opening, as shown for rhabdastin D (**175**) in Scheme 8 [66].

**Figure 20:**
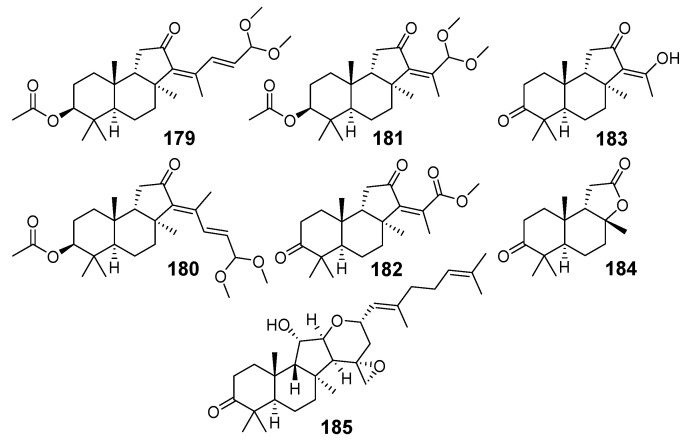
Structures of rhabdaprovidines A–G (**179**–**185**) from *R. providentiae*.

A Vietnamese team studied the sponge *Rhabdastrella providentiae* collected by scuba at the sea area of Con Co (Vietnam) in 2016. Chemical investigation of the methanol extract of this sponge led to the isolation of a series of isomalabaricane *nor-*terpenoids named as rhabdaprovidines A–G (**179**–**185**) [67,68], with structures established by HR-ESI-MS, 1D, and 2D NMR experiments (Figure 20). Moreover, elucidation of the absolute stereochemistry of the most intriguing metabolites, rhabdaprovidines F (**184**) with *β*-configured Me-30 and G (**185**), required an analysis of both experimental and theoretically calculated ECD spectra [68].

**Figure 21:**
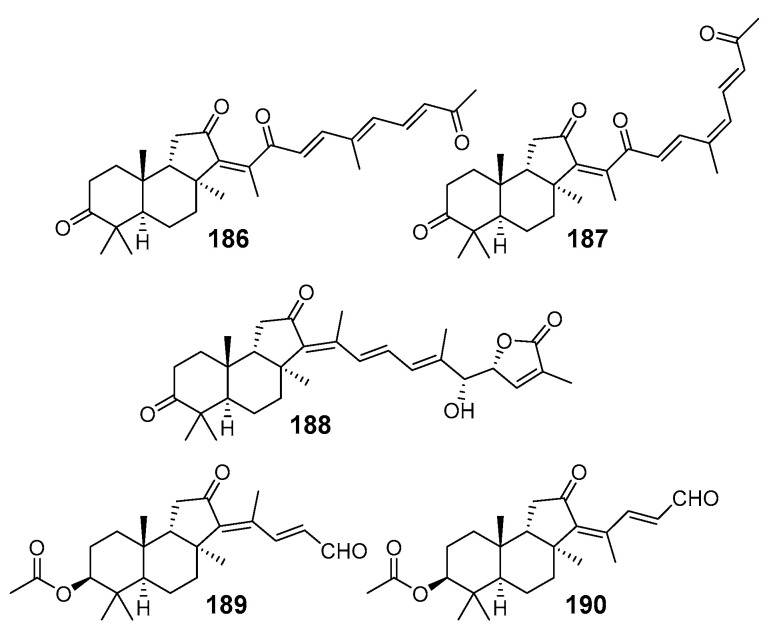
Structures of rhabdastrellins G–K (**186**–**190**) from *R. providentiae*.

Further isolation of the constituents of methanol extract of Vietnamese *R. providentiae* enriched the series of rhabdastrellins A–F (**123**–**128**) from *R.* aff. *distincta* [45] with additional rhabdastrellins G-K (**186**–**190**) [69] (Figure 21). Chemical structures of compounds **186**–**190** were determined by HR-ESI-MS, NMR spectroscopy, experimental and calculated circular dichroism spectra. The isolated rhabdastrellins were evaluated for their cytotoxicity toward HepG2, LU-1, MCF-7, HL-60, and SK-Mel2 human cancer cell lines. Among them rhabdastrellin H (**187**) exhibited significant cytotoxicity, with IC_50_ values ranging from 11.2 ± 1.4 to 16.0 ± 2.0 μM. It is interesting that 20(22)*E*-isomer, rhabdastrellin G (**186**), was not active in this bioassay [69].

In their turn, rhabdaprovidines A–C (**179**–**181**) were evaluated for their anti-inflammatory activity by measuring NO production in LPS stimulated BV2 cells. In this test compounds **179** and **180** exhibited inhibitory activity with IC_50_ values of 20.4 ± 1.5 and 17.5 ± 0.9 μM, respectively [67].

**Figure 22:**
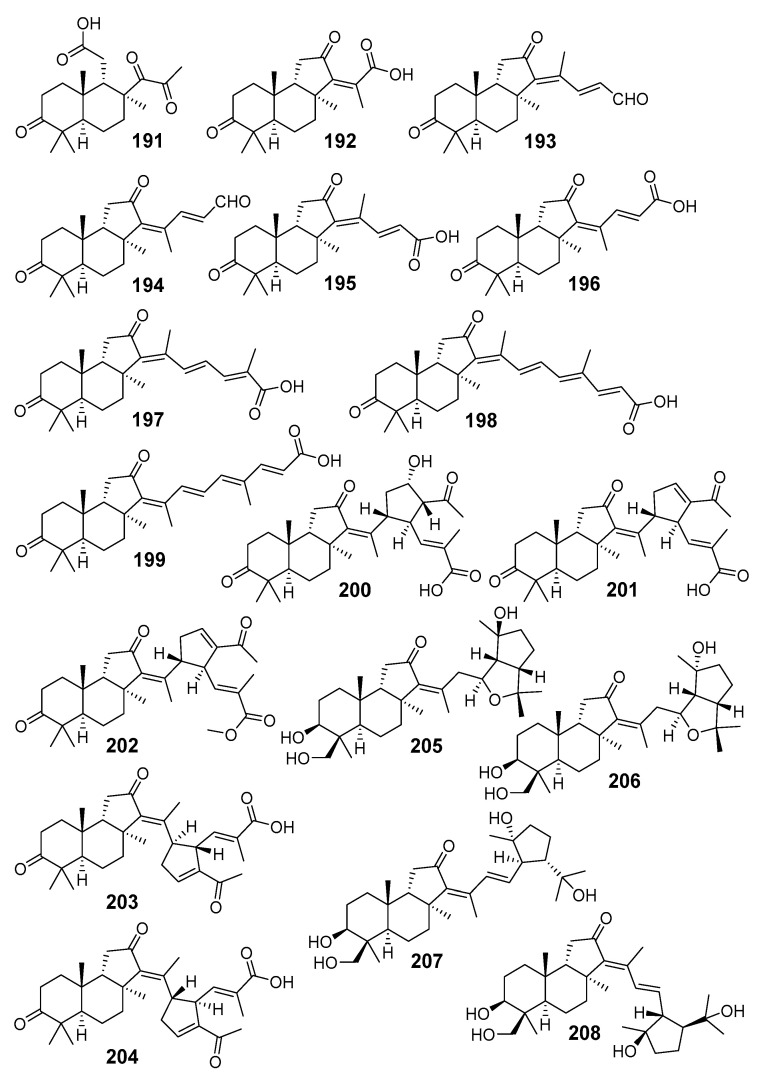
Structures of globostelletins A–R (**191–209**) from *R. globostellata*.

Globostelletins A–I (**191**–**199**) (Figure 22), nine cytotoxic isomalabaricane natural products, were isolated together with rhabdastrellic acid A (**129**), stellettins C-E (**58**–**60**), and jaspolid F (**154**) from the sponge *Rhabdastrella globostellata* collected off coral reefs of Hainan Island (China). The action of these compounds on apoptosis in tumor cells and ubiquitin-proteasome system was studied [44].

Further investigation of this sponge sample gave globostelletins J–R (**200**–**208**) (Figure 22), including unusual derivatives with cyclopentane rings in their side chains. Isolated isomalabaricanes **200**–**208** were tested for the inhibitory activities against human tumor-related protein kinases [70].

Recently, the absolute configurations of the C-15 and C-23 asymmetric centers in the structures of globostelletins M (**203**) and N (**204**) were reassigned according to the NMR and ECD data of these two compounds and their stereoisomers stellettins Q (**74**) and R (**75**) from the Vietnamese sponge *Stelletta* sp. [42].

**Figure 23:**
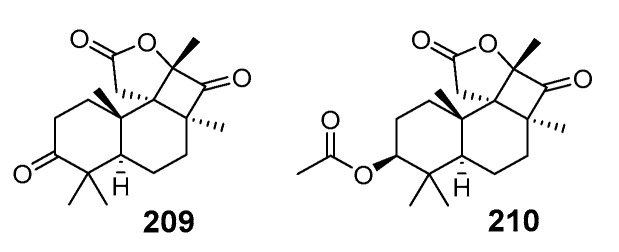
Structures of cyclobutastellettolides A (**209**) and B (**210**) from *Stelletta* sp.

Unusual C_19_ *nor*-terpenoids, cyclobutastellettolides A (**209**) and B (**210**) (Figure 23), were isolated from a *Stelletta* sp. marine sponge collected off Cham Island during the 38th Russian-Vietnamese marine expedition onboard the research vessel “Akademik Oparin” in 2010. The frozen sponge was chopped and extracted with ethanol for further isolation of individual substances. Both compounds with an unprecedented carbon skeleton were structurally elucidated using extensive NMR, MS, and ECD analysis together with quantum chemical modeling and biogenetical considerations.

**Scheme 9:**
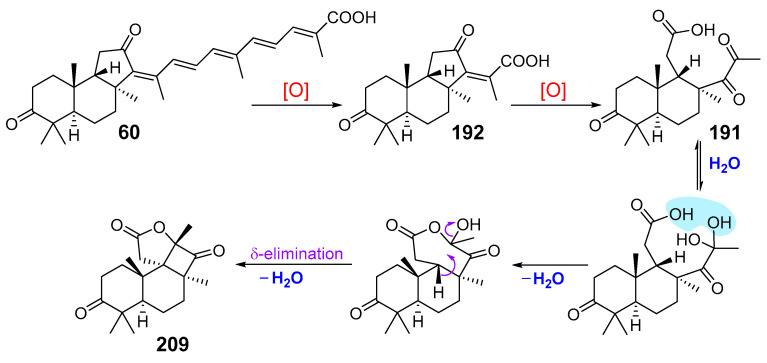
Hypothetical pathway of cyclobutastellettolide A (**209**) biogenesis.

Proposed biogenesis of these compounds as products of stellettin E (**60**) transformation through globostellettin B (**192**) is shown in Scheme 9. Calculation of ΔG for possible intermediates of conversion **192** into **209** suggested the formation of a seven-membered ring, but not a five-membered ring at the first stage of this conversion [71].

Thus, there is a great diversity of isomalabaricane-type triterpenoids and compounds derived from them. While three reports on isolation of isomalabaricanes in special sunlight-protected conditions gave core-oxygenated stellettins J (**64**) and K (**65**) [35], a series of stelliferins **86**–**88** with 29-hydroxy groups, as well as with 22-acetoxy/hydroxy groups [48], and three 3-*epi*-29-hydroxystelliferins **89**–**91** [49], we may conclude that oxygenation in isomalabaricanes could represent not only a degradation process, but also a direction of biosynthetic transformations.

Moreover, rare findings allowed us to presume that symbiotic microorganisms in the corresponding sponges are involved in the generation of some metabolites. Thus, as a result of the chemical investigation of the sponge *Stelletta tenuis*, Li et al. identified two naturally occurring α-pyrones, namely gibepyrones C and F, along with three stellettins N-P (**71**–**73**) [40]. These α-pyrones were supposed to be the oxidation products of the co-occurring isomalabaricane-type triterpenoids [41]. Gibepyrone F had previously been isolated from the fungal plant pathogen *Gibberella fujikuroi* [72], as well as from the sponge *Jaspis stellifera* [63].

However, some sponge metabolites belonging to the isomalabaricane class, especially representatives of *nor*-terpenoids, are likely artificial products formed by destroying genuine triterpenoid structures.

Nevertheless, isomalabaricane-type artificial compounds are also of great interest because many of them exhibit biological activities almost of the same level as related triterpenoids. The relationship between structures and, for example, cytotoxicity against tumor cells could be established in studies on both isolated triterpenoids and *nor-*terpenoids. Finally, isolation and structure determination of all products could help to understand detailed mechanisms of photo-induced processes on isomalabaricane triterpenoids in frozen, lyophilized, and/or solvents-treated invertebrates, as well as their conversions by sponge enzymes.

### 3.2. Biogenesis, Taxonomic Distribution, and Biological Activities of Isomalabaricanes

**Scheme 10:**
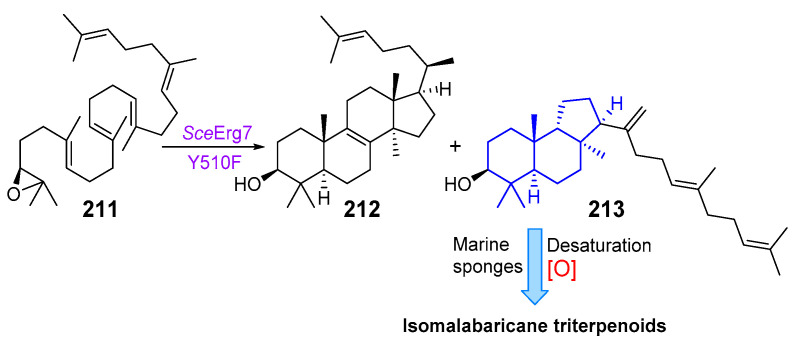
Biogenesis of isomalabaricanes by the mutant lanosterol synthase.

The cyclization of squalene to the precursor of malabaricane triterpenoids by mutant squalene–hopene cyclase [26] or acid catalysis [5] has already been mentioned in this review. Isomalabaricanes are undoubtedly products of unusual cyclization of 2,3-oxidosqualene (**211**). Lodeiro et al. described Erg7 Tyr510 mutants of lanosterol synthase from fungi that, in addition to lanosterol (**212**), also generates a novel triterpenoid, (13*α*H)-isomalabarica-14(27),17E,21-trien-3*β*-ol (**213**). This product was isolated and identified by NMR spectroscopy. Triterpenoid **213**, which has a *trans*–*syn*–*trans* rings junction in the tricyclic core, is a likely biosynthetic precursor of isomalabaricane triterpenoids in sponges. It was suggested that sponges could cyclize 2,3-oxidosqualene (**211**) into **213** and then convert it into various isomalabaricane terpenoids by desaturation and specific oxidation (Scheme 10). The enzyme that constructs **213** in sponges is likely evolved from a sponge lanosterol synthase [73].

The taxonomic distribution of sponges as producers of isomalabaricane triterpenoids and related compounds is very limited. All the demospongiae sponges capable of accumulating these triterpenoids and compounds derived from them belong to the order Tetractinellida and were collected in tropical and/or subtropical waters [1,2,41]. Generally, isomalabaricanes were isolated from sponges identified as representatives of only four genera*: Jaspis*, *Stelletta*, *Rhabdastrella*, and *Geodia*. However, taxonomic identification of many above-mentioned animals remains questionable. Kennedy showed that sponges belonging to the so-called “*Jaspis stellifera* complex”, used for the isolation of isomalabaricane-type compounds, are really different specimens of *Rhabdastrella globostellata.* As possession of malabaricane-type triterpenes was determined to be a good chemotaxonomic marker for Stellettids, it was also suggested that *J. stellifera* from Japanese waters are also misidentified specimens of *S. globostellata* since they too have been reported to contain malabaricane-type triterpenes [74].

Concerning sponges of the genus *Stelletta*, it was noticed that their secondary metabolites are quite different depending on the collection. Indeed, isomalabaricane triterpenoids were mainly found as very complex mixtures in tropical sponge samples, while boreal and cold-water sponges primarily contain alkaloids and lipids. From a chemo-ecological point of view, this indicates that the studied sponges are able to produce different types of secondary metabolites in order to adapt to the various living conditions [41]. Otherwise, it might evidence the misidentification of some sponge samples. Naturally, doubts about the correctness of the species assignments do not apply to isolated substances and their structures.

The biological activities of isomalabaricane-type compounds are very impressive. The extremely high cytotoxicity against tumor cells has attracted the attention of many scientific groups [75], including the National Cancer Institute (Frederick, MD, USA) [48]. In fact, stellettin A (**57**) showed an IC_50_ of 2.1 nM against murine leukemia P388 cell line [32,75]. More recently, Liu et al. established that the apoptotic properties of stellettins A (**57**) and B (**43**), as well as that of geoditins A (**130**) and B (**131**), are associated with the induction of oxidative stress in tumor cells. They also indicated an important role of a ketone group at C-3 for free radical production and dissipation of mitochondrial membrane potential, that causes apoptosis of leukemia HL-60 cells. Among the isomalabaricanes studied by them, geoditin A with IC_50_ < 6.6 µM was the most promising [76].

During continued investigation, stellettin A (**57**) showed quite different cytotoxicity against human leukemia HL-60 cells (IC_50_ 0.4 µg/mL) and human prostate cancer LNCaP cells (IC_50_ 120 µg/mL). The dramatic increase in generation of reactive oxygen forms, dissipation of mitochondrial potential, and apoptosis through a FasL-caspase-3-apoptotic pathway were revealed as characteristic features of action of this isomalabaricane derivative against tumor cells. HL-60 cells were more sensitive than LNCaP cells by an order of magnitude [77].

The same compound **57** inhibited the growth of B16F10 murine melanoma cells by the induction of endoplasmic reticulum stress and accumulation of unfolded proteins, including abnormally glycosylated melanoma marker proteins, tyrosinase and tyrosinase-related protein 1. It increased a level of autophagosome-associated protein light chain 3 in a membrane-bound form. Therefore, stellettin A is an endoplasmic reticulum stressor, inhibitor of melanoma cells growth, and inductor of autophagy of these cells [78].

Recent studies on the action of stellettin B (**43**) on 39 human cancer cell lines showed its significant cytotoxic effect against the human glioblastoma cell line SF295 with a GI_50_ value of 0.01 μM. Cytotoxic activity against normal human cell lines such as HMEC, RPTEC, NHBE, and PrEC revealed GI_50_~10 μM, demonstrating its selective toxicity against cancer cells in comparison with normal lines. Compound **43** inhibited the phosphorylation of Akt, without any activity toward p-ERK and p-p38, indicating that inhibition of PI3K/Akt pathway might be involved in the effects of this compound on tumor cells. Moreover, as the homogenous time-resolved fluorescence assay showed that stellettin B (**43**) did not inhibit PI3K activity, the direct target might be a signal protein upstream of Akt pathway other than PI3K [79]. In addition, stellettin B influences activity of vascular endothelial growth factor (VEGF) in glioblastoma cells and exhibits anti-invasion and antiangiogenic effects by inhibition of VEGF and Akt/Girdin signaling pathway [80]. This suggests **43** to be a promising anticancer agent for glioblastoma therapy [80,81].

Another study on stellettin B (**43**), isolated from the marine sponge *Jaspis stellifera,* demonstrated that this compound induced G1 arrest, apoptosis, and autophagy at low concentrations in human non-small lung cancer (NSCLC) A549 cells. These processes were triggered by targeting PI3K/Akt/mTOR pathway, while cell arrest by stellettin B may be induced by the reduction of cyclin D1 and enhancement of p27 expression [82].

Stellettin B inhibits proliferation of human tumor cells K562 and KU812 with IC_50_ of 0.035 µM and 0.95 µM, respectively. No obvious cell cycle arrest was observed; apoptosis was induced in K562 cells after stellettin B treatment. Inhibition of Stat5 and PI3K might be involved in this process via suppression of Stat5 phosphorylation, inhibition of the expression of 4PI3K catalytic isoforms, and phosphorylation of downstream effectors PDK1 and Akt. In addition, a synergistic effect of stellettin B and imatinib was found, which made stellettin B a promising candidate against chronic myeloid leukemia therapy either alone or together with imatinib [83]. According to reports mentioned above and some other observations concerning both compounds [52], molecular mechanisms of stellettins A and B action are different, and therefore two possible configurations of the 13-double bond alter the binding sites of these compounds.

Stelletin B (**43**) and (–)-stellettin E (**60**) were selectively active toward p21^WAF1/Cip1^-deficient human tumor cells HCT-16 with IC_50_ values of 0.043 µM and 0.039 µM, respectively [34]. Some other stellettins were less toxic against this and other tumor cell lines.

In their turn, stelliferin riboside (**101**) and 3-O-deacetyl-13(*Z*)-stelliferin riboside (**102**) were extremely toxic against the L5178Y mouse lymphoma cell line with IC_50_ values of 0.22 nM and 2.4 nM, respectively. Compound **102** also showed moderate activity toward *Escherichia coli*, exhibiting inhibition zones of 12 mm at a loading concentration of 10 µg [53].

Other isomalabaricanes from sponges may also lead to oxidative stress and apoptosis in human tumor cells. For example, rhabdastrellic acid A (**129**) at nanomolar concentration (IC_50_ = 1.46 nM) significantly downregulated the phosphorylation of P85/PI3K and its downstream target Akt and induced caspase-3-dependent apoptosis in HL-60 cells [84].

Geoditin A (**130**) induced an apoptosis associated with a decrease in transferrin receptors expression and oxidative stress in colon cancer HT29 cells. As the apoptosis was diminished by pre-treatment with oxidant scavenger, N-acetylcysteine, the apoptosis-inducing activity of geoditin A is likely mediated through oxidative stress [85]. In case of murine melanoma B16 cells, geoditin A (**130**) not only initiated oxidative stress and apoptosis, but also decreased expression of melanogenic proteins and cell melanogenesis. Taking into account its potent antimelanogenic property and relatively low cytotoxicity as well as a capability to inhibit tyrosinase activity at sublethal doses, geoditin A demonstrated therapeutic potential as a perspective skin lightening agent [86].

The effect of jaspolide B (**150**) from *Jaspis* sp. on hepatoma cells was studied and this compound moderately inhibited the growth of Bel-7402 and HepG2 cells with IC_50_ values of 29.1 µM and 29.5 µM, respectively. Compound **150** induced apoptosis of these cells and arrested the cell cycle progression at G1 phase. Moreover, it caused the dose-dependent disassembly of microtubule cytoskeleton in Bel-7402 cells, but this effect was weaker than that of colchicine. The multiple mechanisms of the cytotoxic action of this compound against human hepatoma cells might be taken into account in further investigations of this and related compounds as potential anticancer agents [87].

Eight globostellatic acid congeners (**109**–**116**) from the sponge *Rhabdastrella globostellata* were highly active against the mouse melanoma tumor cells L5178Y. Some of them also showed moderate antibacterial properties, but did not demonstrate potent antifungal activity [51,53].

There are other reports about less potent cytotoxic action of isomalabaricane-type compounds. However, extremely high activities of stellettins A and B as well as of some other close-related compounds are attractive for their application. The main reason that inhibits their clinical testing and further development as antitumor drugs is possibly their sensitivity to sunlight.

Uncontrolled angiogenesis is a pathological process, often associated with such diseases as atherosclerosis, arthritis, diabetic retinopathy, and cancer. The growth of solid tumor requires increase in vascularization. Globostellatic acid X methyl esters with 13*E*-geometry of a double bond (**119** and **120**) selectively inhibit the proliferation of human umbilical vein endothelial cells and, therefore, are promising agents for cancer therapy [57].

Stellettin G (**41**) from the Hainan sponge *Stelletta s*p. was found to be a potent inhibitor of protein-tyrosine phosphatase 1B (PTP1B) with IC_50_ = 4.1 ± 0.9 µM [37].

Neuroprotective effects of isomalabaricanes were also reported. During the search for pharmaceutical agents halting the progression of Parkinson’s disease, Taiwanese scientists have found the capability of stellettin B (**43**) to protect SH-SY5Y cells against 6-OHDA-induced cellular damage. SH-SY5Y is a thrice-subcloned cell line, derived from the SK-N-SH neuroblastoma cells, which is used as a model for neurodegenerative disorders. In its turn, 6-hydroxydopamine (6-OHDA) is a hydroxylated analogue of dopamine that has been exploited in experimental model of Parkinson’s disease as inductor of cellular damage and oxidative stress through PI3K/Akt, MAPK, caspase cascade and Nrf2/HO1 cascade modulation. Compound **41** reversed a locomotor deficit in an in vivo experiment, using the zebrafish model of Parkinson’s disease, demonstrating a potential for the developing of a drug candidate for PD treatment [88].

The anti-inflammatory effect of rhabdaprovidines A–C (**179**–**181**), isolated from the Vietnamese sponge *Rhabdastrella providentiae*, was investigated by measuring NO production in LPS-stimulated microglial BV-2 cells. Compounds **179**–**181** inhibited NO production with IC_50_ values of 20.4 ± 1.5, 17.5 ± 0.9, and 46.8 ± 2.3 µM, respectively [67].

## 4. Syntheses of Isomalabaricane-Type Compounds

**Scheme 11:**
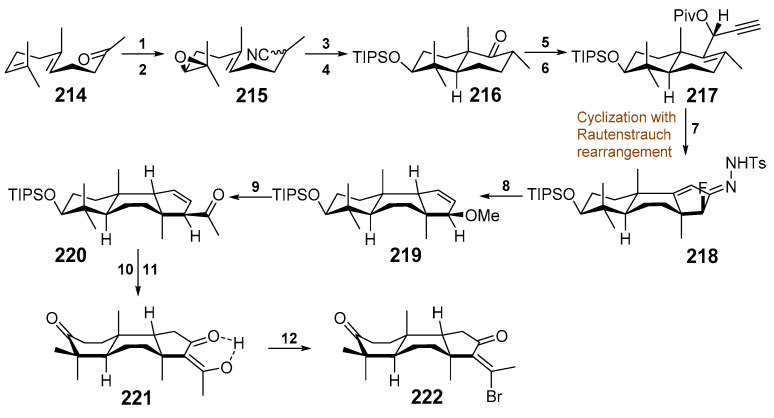
Synthesis of the core moiety of isomalabaricane terpenoids. Reagents and conditions: 1. TosMIC, t-BuOK, 94%; 2. NBS, THF/H_2_O, then K_2_CO_3,_ 82%; 3. Cp_2_TiCl_2_, Zn, 70%; 4. TIPSOTf, 95%; 5. LDA, CH_2_Cl_2_, then LiClO_4_, 80%; 6. C_2_H_2_, *n*-BuLi, then PivCl, 82%; 7. [Au(PPh_3_)Cl], AgOTf, Selectfluor, then NH_2_NHTs, 81%; 8. MeOH, Et_3_N, then CatBH, CsOAc, 58%; 9. Cp_2_ZrCl_2_, *n*-BuLi, then CuOAc, AcCl, 64%; 10. BH_3_, TfOH, then H_2_O_2_; 11. IBX, 80% over 2 steps; 12. (COBr)_2._, DMF.

In spite of a significant interest in potent cytotoxic action of isomalabaricanes against tumor cells, their synthesis was described only very recently. Scientists from Roger Adams Laboratory of Illinois University (IL, USA) reported total syntheses of (±)-rhasbdastrellic acid and (±)-stellettin E [89]. For this purpose, they used a linear sequence of fourteen steps from commercial geranyl acetone (**214**). The difficult task of construction of strained *trans*–*syn*–*trans-*perhydrobenz(e)indene core of isomalabaricane triterpenoids was accessed in selective manner using oxidative Rautenstrauch cycloisomerization to yield the ring C with the desired configuration at C-8 and ring B in the boat conformation (Scheme 11).

Boyko et al. carried out eight steps to convert geranyl acetone (**214**) into the derivative **222**, containing all the stereochemical peculiarities characteristic of core moiety of isomalabaricane triterpenoids. More than five grams of the intermediate **217** as a single diastereomer were obtained to facilitate further steps of the synthesis. Another product in this sequence of transformations, namely **219**, was also accumulated in the yield on gram scale. The last intermediate compound **221** was studied by X-ray analysis, which showed that ring A also has a bath conformation. Bromoketone **222** was used as a starting compound for construction of target rhabdastrellic acid A (**129**) and stellettin E (**60**).

**Scheme 12:**
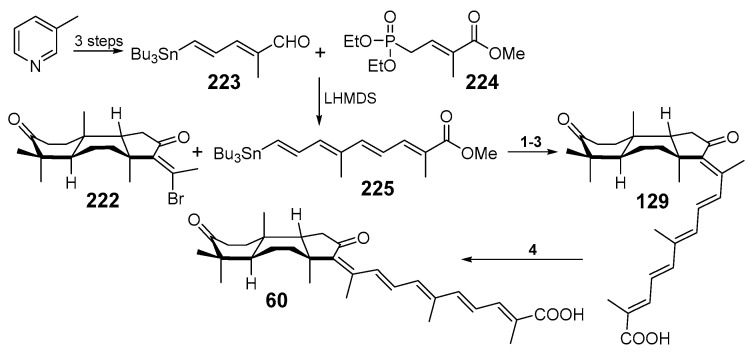
Synthesis of methyl esters of rhabdastrellic acid A (**226**) and stellettin E (**227**). Reagents and conditions: 1. Pd_2_(dba)_3_, Ph_3_As, 45%; 2. Me_3_SnOH, 98%; 3. LiOH, 96%; 4. *h*ν, 400 nm, 53% after two cycles.

Having the fully oxidized core of the isomalabaricanes, the syntheses of racemic rhabdastrellic acid A and racemic stellettin E were completed by cross coupling. The coupling partner was synthesized from 3-picoline into three steps to give stannate **223** followed by the reaction with phosphonate **224**. As a result, the obtained polyene stannate **225** was used as a coupling partner in the reaction with bromoketone **222** and gave the methyl ester of rhabdastrellic acid A in three steps (45% overall yield). Saponification of the ester with trimethyltin hydroxide quantitatively led to racemic rhabdastrellic acid A (**129**), identical to the reported natural metabolite. Compound **129** was converted into methyl ester of stellettin E (**227**) by known olefin 13-*Z*/*E*-photoisomerization after irradiation by visible light (Scheme 12).

## 5. Conclusions

So far, there have been no review articles exclusively devoted to different aspects of the studies on tricyclic malabaricane and isomalabaricane triterpenoids, although about two hundred compounds belonging to this structural group are described in literature.

In spite of a great diversity of known isomalabaricanes, these compounds, as well as malabaricanes, remain rare natural products. According to the present data, the taxonomic distribution of their producers is limited to only a few species of higher plants, ferns, and fungi in the case of malabaricanes, and a small number of marine sponge species for isomalabaricanes. Geographically, these producers inhabit mainly tropical and subtropical terrestrial areas and tropical sea waters, respectively.

The extremely high cytotoxicity of some isomalabaricanes against tumor cells and other biological activities has attracted attention, but none of these compounds have yet been developed as a drug. One of the substantial reasons for this is their instability, particularly the tendency to photoisomerization of the 13-double bond. We believe that forthcoming studies on biogenesis of these unusual metabolites will allow a better understanding of the peculiarities of triterpenes evolution and the obtaining of new enzymes involved in their biosynthesis. Moreover, synthetic approaches promise future progress in biomedical research and can likely lead to the selection of new drug candidates and provide their availability in sufficient quantities.

We hope that this review will help scientists from different countries interested in these substances in the search for new metabolites of this class and study of their properties, including biological action. We would also be glad if it inspires new specialists to join the work with these promising compounds.

## Figures and Tables

**Table 1:** Taxonomic positions of biological sources of malabaricane triterpenoids.

Compounds	Biological Source				
	Kingdom	Class/Clade	Order	Family	Species
**1**–**5**, **8–11**	Plantae	Angiosperms	Sapindales	Simaroubaceae	*Ailanthus tryphysa* *(=malabarica)*
**6**,**7**	Plantae	Angiosperms	Malpighiales	Flacourtiaceae	*Caloncoba echinata*
**16**,**17**	Plantae	Angiosperms	Sapindales	Burseraceae	*Bursera microphylla*
**18**,**19**	Plantae	Spermatophyta	Asterales	Asteraceae	*Pyrethrum* *santolinoides*
**18**,**22**	Plantae	Pistacia	Sapindales	Anacaridiaceae	*Pistacia lentiscus*
**24**–**26**	Plantae	Magnoliopsida	Fabales	Fabaceae	*Adesmia* *aconcaguensis*
**27**–**32**	Mycota	Basidiomycota	Agaricales	Cortinariaceae	*Cortinarius* *arcuatorum,* *C. sadagnitus,* *C. fulvoincarnatus*
**34**,**35**	Plantae	Tracheophyta	Polypodiales	Adiantaceae	*Lemmaphyllum* *microphyllum* var. *obovatum*
**38**	Plantae	Tracheophyta	Polypodiales	Polypodiaceae	*Colysis elliptica*
**35**	Unknown	Bottom sediment, Lake Cadagno (Switzerland)			

## Data Availability

Data sharing is not applicable to this article.
